# Key to High Performance Ion Hybrid Capacitor: Weakly Solvated Zinc Cations

**DOI:** 10.1002/advs.202305532

**Published:** 2023-11-23

**Authors:** Peng Chen, Xiaohan Sun, Bernd Plietker, Michael Ruck

**Affiliations:** ^1^ Faculty of Chemistry and Food Chemistry Technische Universität Dresden 01062 Dresden Germany; ^2^ Max Planck Institute for Chemical Physics of Solids Nöthnitzer Straße 40 01187 Dresden Germany

**Keywords:** capacitors, coulombic efficiency, electrolytes, solvent effect, zinc

## Abstract

Zinc ion hybrid capacitors suffer from lack of reversibility and dendrite formation. An electrolyte, based on a solution of a zinc salt in acetonitrile and tetramethylene sulfone, allows smooth zinc deposition with high coulombic efficiency in a Zn||stainless steel cell (99.6% for 2880 cycles at 1.0 mA cm^−2^, 1.0 mAh cm^−2^). A Zn||Zn cell operates stably for at least 7940 h at 1.0 mA cm^−2^ with an area capacity of 10 mAh cm^−2^, or 648 h at 90% depth of discharge and 1 mA cm^−2^, 9.0 mAh cm^−2^. Molecular dynamics simulations reveal the reason for the excellent reversibility: The zinc cation is only weakly solvated than in pure tetramethylene sulfone with the closest atoms at 3.3 to 3.8 Å. With this electrolyte, a zinc||activated‐carbon hybrid capacitor exhibits an operating voltage of 2.0 to 2.5 V, an energy‐density of 135 Wh kg^−1^ and a power‐density of 613 W kg^−1^ at 0.5 A g^−1^. At the very high current‐density of 15 A g^−1^, 29.3 Wh kg^−1^ and 14 250 W kg^−1^ are achieved with 81.2% capacity retention over 9000 cycles.

## Graphical abstract

An optimized Zn hybrid electrolyte assures dendrite‐free, reversible zinc plating and stripping at capacity densities up to 50 mAh cm^−2^ and discharge depths of 90%. A Zn||activated‐carbon ion capacitor with this electrolyte operates at 2.0 to 2.5 V and achieves a capacity retention of 91% after 5000 cycles, an energy density of 29.3 Wh kg^−1^ with a power density of 14250 W kg^−1^ at a current density of 15 A g^−1^.

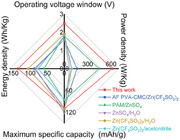

## 1 Introduction

Electrochemical double‐layer capacitors, often denoted as supercapacitors, are attracting extensive attention.^[^ ^1^ ^]^ In double‐layer capacitors, the energy is supplied by physically adsorbing charged ions at the interface between the electrodes and the electrolyte (electrochemical double layer), which enables kinetically fast transfer of charged ions.^[^ ^2^ ^]^ The absence of Faradaic ion intercalation reactions as in parallel connected batteries, gives double‐layer capacitors the advantage of fast charging/discharging rates, high efficiency, and long cycle life.^[^ ^3^ ^]^ This is accompanied by a predictable lower energy density, typically below 10 Wh kg^−1^ at 0.05 A g^−1^ in aqueous electrolytes.^[^ ^2^, ^4^ ^]^ Using organic electrolytes, double‐layer capacitors can operate at 2.7 V and 5–8 Wh kg^−1^ at a constant power discharge of 317 W kg^−1^.^[^ ^5^ ^]^ Operating voltage windows of 3–4 V and energy densities of 16 or 116 Wh kg^−1^ at 2.0 or 0.2 A g^−1^ have been reported by using ionic liquid electrolytes, such as 1‐ethyl‐3‐methylimidazolium tetrafluoroborate^[^ ^6^, ^7^ ^]^ or mixture with tetramethylammonium tetrafluoroborate.^[^ ^8^ ^]^ Unfortunately, the high viscosity of ionic liquids and the resulting unsatisfactory kinetics have become a bottleneck for their further development.^[^ ^9^, ^10^, ^11^, ^12^ ^]^

Zinc ion batteries have flourished attributed to the merits of Zn electrodes such as high specific capacity (820 Ah kg^−1^), low electrochemical potential (−0.762 V vs standard hydrogen electrode), low cost, abundance, environmental friendliness, and good compatibility with aqueous or organic electrolytes.^[^ ^1^ ^]^ Rechargeable zinc ion batteries have diverse cathode materials, which can be mainly classified as manganese or vanadium‐based oxides, Prussian blue analogues, spinel‐type oxides, layered sulfides, Chevrel phases, polyanionic compounds, organic materials, and a few others materials.^[^ ^13^ ^]^ Manganese‐based oxides, such as MnO_2_, Mn_2_O_3_, and Mn_3_O_4_, have attracted great interest in electrochemical energy storage because of their high natural abundance and the rich redox chemistry of Mn. In particular, MnO_2_ is useful due to its high specific capacity of 308 mAh g^−1^.^[^ ^13^ ^]^

In terms of electrolytes, besides the most widely used aqueous and organic electrolytes, there are also ionic liquids, deep eutectic and solid‐state electrolytes. To address the problems of water decomposition and zinc dendrite formation in aqueous electrolytes, several strategies were presented, such as high‐concentration electrolytes or organic solvent additives. Specifically, mixtures of aqueous electrolytes and organic solvents, such as dimethyl carbonate,^[^ ^14^ ^]^ propylene carbonate,^[^ ^15^ ^]^ acetonitrile (AN, CH_3_CN),^[^ ^16^ ^]^ triethyl phosphate,^[^ ^17^ ^]^ dimethyl sulfoxide,^[^ ^18^ ^]^ ethylene glycol,^[^ ^19^ ^]^ or methanol,^[^ ^20^ ^]^ have also been widely explored. Nevertheless, zinc ion batteries still suffer from limited power density, short cycling life and safety issues.^[^ ^21^, ^22^ ^]^

Hybrid capacitors combining capacitor‐based and battery‐based electrodes are considered as more promising devices.^[^ ^23^ ^]^ The working mechanism of the zinc ion hybrid capacitor is based on reversible ion adsorption/desorption on the cathode and Zn^2+^ deposition/stripping on the zinc anode. During charging, [NTf_2_]^−^ anions will be absorbed into activated carbon and Zn^2+^ will get electrons deposited on the other side of zinc electrode. During discharging, [NTf_2_]^−^ anions will be desorbed from activated carbon and Zn will lose electrons striped on the other side of zinc electrode into the electrolyte.^[^ ^24^ ^]^

Diverse Zn ion hybrid capacitors, consisting of a carbon cathode and a Zn anode (with respect to discharge process), have been developed. Yet, their practical application still faces many challenges. First, in diluted aqueous Zn ion electrolytes (pH 4–5), unwanted H_2_ generation and irreversible by‐products, such as Zn_4_(OH)_6_SO_4_·*x*H_2_O or Zn(OH)_2_, exacerbate the corrosion of the Zn electrode and only low coulombic efficiency (the ratio of charge to discharge capacity, CE) can be achieved. Second, the formation of dendrites during the plating/stripping processes not only limits the cyclic stability of Zn electrodes, but also leads to safety problems.^[^ ^1^ ^]^ Finally, most Zn ion hybrid capacitors operating in aqueous electrolytes have rather limited operating voltages (typically 0.2 to 1.8 V) in order to minimize the competitive evolution of H_2_ (<0.2 V vs Zn^2+^/Zn) or O_2_ (>1.8 V vs Zn^2+^/Zn) of water.^[^ ^2^ ^]^ Accordingly, the design of electrolytes with a wide electrochemical window and high Zn plating/stripping efficiency while ensuring uniform Zn deposition is key to the development of high‐performance Zn ion hybrid capacitors.

Based on our recent work on solutions of metal cations in ionic liquids,^[^ ^25^, ^26^ ^]^ we propose a Zn ion hybrid capacitor with an optimized electrolyte that is a solution of zinc di[bis(trifluoromethylsulfonyl)imide] (Zn[NTf_2_]_2_, Zn[TFSI]_2_, Zn[TFSA]_2_, Zn((CF_3_SO_2_)_2_N)_2_) in a mixture of anhydrous acetonitrile and tetramethylene sulfone (TMS, sulfolane, C_4_H_8_O_2_S). This new electrolyte combines low viscosity, which provides good transport kinetics, with good oxidation stability, which enables high operating voltage. In addition, a special feature of the electrolyte facilitates smooth deposition and a strong affinity with the Zn(001) surface, enabling dendrite‐free Zn plating/stripping with a very high area capacity (50 mAh cm^−2^). In this way, we achieve Zn ion hybrid capacitors with high operating voltage, high current and power density, high utilization and long cycle stability with satisfactory CE.

## 2 Results and Discussion

### 2.1 Electrochemical Characterization of the Zn Electrodes

**Figure 1:**
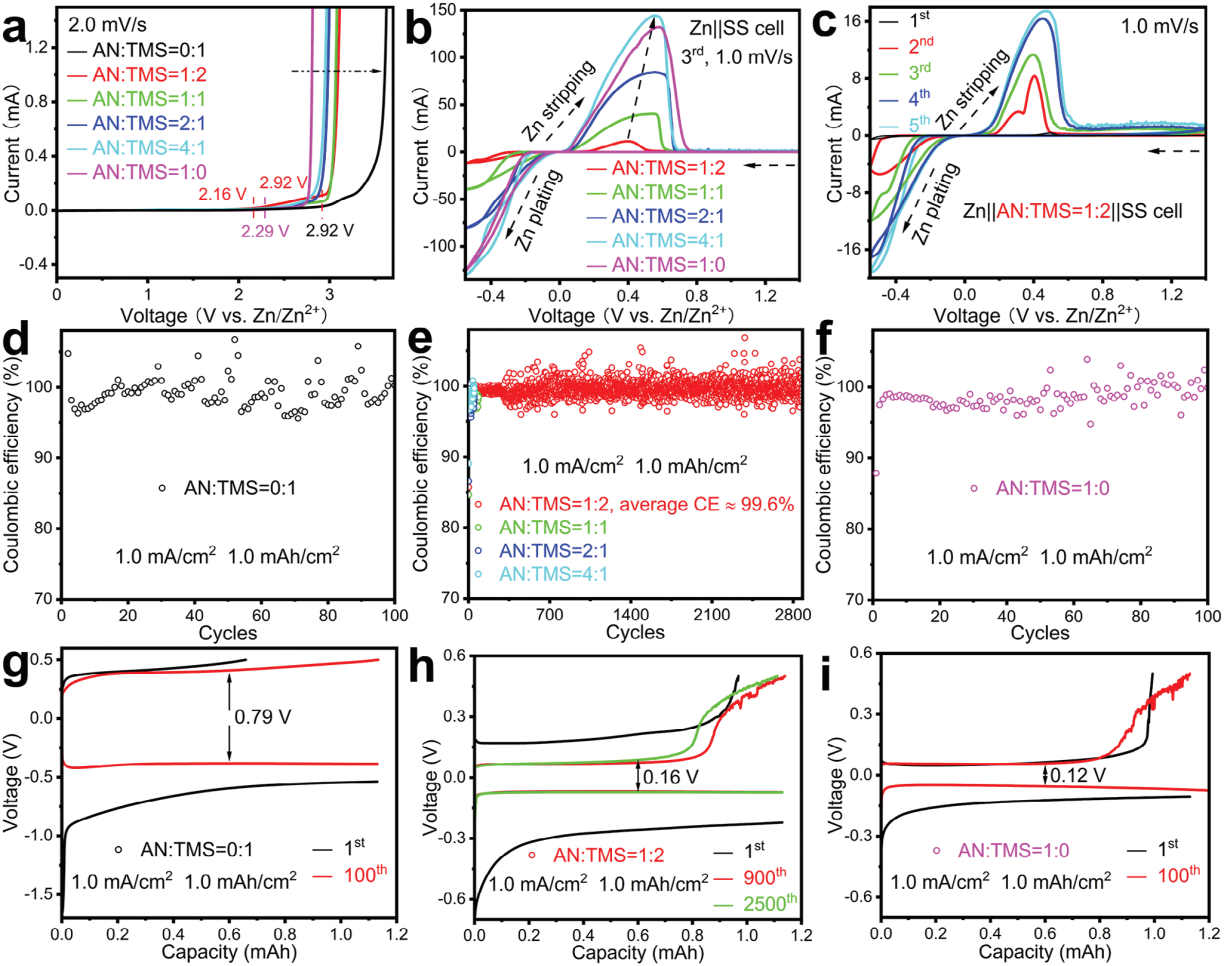
The oxidation stability and coulombic efficiency of 0.5 m Zn[NTf_2_]_2_/(*V*_AN_ : *V*_TMS_ = 0:1, 1:2, 1:1, 2:1, 4:1, and 1:0) electrolytes in a Zn||SS cell. a) LSV curves and b) CVs. c) The first five CV cycles with 0.5 m Zn[NTf_2_]_2_/(*V*_AN_ : *V*_TMS_ = 1:2). d–f) Coulombic efficiency at 1.0 mA cm^−2^ and 1.0 mAh cm^−2^ (discharge: Zn plating at SS electrode, charge: Zn stripping from SS electrode), and (g–i) corresponding voltage profiles of selected cycles. d,g) 0.5 m Zn[NTf_2_]_2_/TMS. e,h) 0.5 m Zn[NTf_2_]_2_/(*V*_AN_ : *V*_TMS_ = 1:2, 1:1, 2:1, and 4:1). f,i) 0.5 m Zn[NTf_2_]_2_/AN.

The yellowish TMS and the colorless AN are readily miscible at various volume ratios (*V*_AN_ : *V*_TMS_ = 1:2, 1:1, 2:1, 4:1) and form a clear transparent liquid (Figure S1a, Supporting Information). Zinc acetate Zn(OAc)_2_ and zinc trifluoromethanesulfonate Zn[OTf]_2_ do not dissolve in pure TMS to the extent that 0.5 mol L^−1^ (0.5 m) solutions are obtained (Figure S1b and the leftmost figure in Figure S1c, Supporting Information). In contrast, the Zn[NTf_2_]_2_ salt dissolves completely in pure TMS, AN, or their mixtures and forms stable electrolytes, which is why we chose Zn[NTf_2_]_2_ in subsequent experiments (Figure S1d, Supporting Information). In a mixture with the volume ratio *V*_AN_:*V*_TMS_ = 1:2, Zn[NTf_2_]_2_ has its highest solubility of 3.0 m (Figure S1e, Supporting Information). Linear sweep voltammetry (LSV) curves were measured to test the oxidation stability of the electrolytes (**Figure** **1**a). The onset voltage of the oxidative decomposition is about 2.3 V for a 0.5 m Zn[NTf_2_]_2_/AN solution and 2.9 V for a 0.5 m Zn[NTf_2_]_2_/TMS electrolyte, which is based on the potential at which the curve begins to deviate from the *x*‐axis.^[^ ^27^ ^]^ With increasing TMS content, the oxidation stability of 0.5 m Zn[NTf_2_]_2_ solutions in AN/TMS mixtures increases (Figure 1a). Moreover, the potential for the onset of anodic decomposition at a constant current density of 0.05 mA cm^−2^ is shown in Figure S2a (Supporting Information).^[^ ^28^ ^]^ In comparison, similar aqueous electrolytes are stable only up to 1.8 V.^[^ ^1^ ^]^

Cyclic voltammograms (CV) of Zn||stainless‐steel spacer (SS) cells were measured to evaluate the reversibility and kinetics in these electrolytes (Figure 1b). In all cases, stable reduction (plating) and oxidation (stripping) of Zn was observed. Comparing the current scales in the longitudinal coordinates, it is evident that the kinetics improves with the AN content, which we attribute to decreasing viscosity of the solution (Figure 1b;Figure S2b, Supporting Information). For the Zn||SS cell with 0.5 m Zn[NTf_2_]_2_/(*V*_AN_:*V*_TMS_ = 1:2), the fourth and fifth cycles overlap almost perfectly after the first three cycles of electrode and electrolyte interface stabilization, indicating high electrochemical reversibility (Figure 1c).

The CE was determined using a galvanostatic charge/discharge measurement in the Zn||SS cell. In the case of the 0.5 m Zn[NTf_2_]_2_/TMS electrolyte, its high viscosity and comparatively high melting point (20–26 °C) led to unstable CE (Figure 1d), and after 100 cycles, the overpotential of charge and discharge was still as high as 0.79 V (Figure 1g). On the other hand, using a 0.5 m Zn[NTf_2_]_2_/AN electrolyte, although the stability of CE increased (Figure 1f) and the overpotential was only 0.12 V, voltage fluctuations were found in the charging curve (Figure 1i), which were probably due to an unstable electrolyte‐electrode interface and a slight decomposition of the electrolyte. Although the increase in acetonitrile content led to an enhancement of CE in the initial third cycle (Figure S2c, Supporting Information), a more severe instability was observed during long‐term cycling (Figure 1e and Figure S3a, Supporting Information). In the case of the favored 0.5 m Zn[NTf_2_]_2_/(*V*_AN_:*V*_TMS_ = 1:2) mixed solvent electrolyte, the chronocoulometric curves after stabilizing the interface for the first three cycles, CE gradually improves (Figure S2d, Supporting Information). In tests of different ratios *V*_AN_:*V*_TMS_ of the 0.5 m Zn[NTf_2_]_2_ hybrid electrolytes, the 1:2 mixture showed the highest average CE (99.6% over 2880 cycles; Figure 1e and enlarged image for the first 100 cycles in Figure S3a (Supporting Information); Figure 1h and Figure S3b–e, Supporting Information). In the first cycle of the stabilization process, the CE is 90.8%, and in the second cycle a CE of 99.4% is obtained, all of which illustrate the stability and high reversibility of our electrolyte even with 50% DOD_Zn_ in a Zn||SS cell (Figure S3f, Supporting Information). The high degree of overlap of the 900th and 2500th charge/discharge curves and the stable lower overpotential of 0.16 V are also clear indications of high reversibility provided by the electrolyte (Figure 1c,h). Since the 0.5 m Zn[NTf_2_]_2_/(*V*_AN_:*V*_TMS_ = 1:2) electrolyte offers the best combination of high voltage oxidation stability, sufficient kinetics and high CE, it was selected for the following experiments.

**Figure 2:**
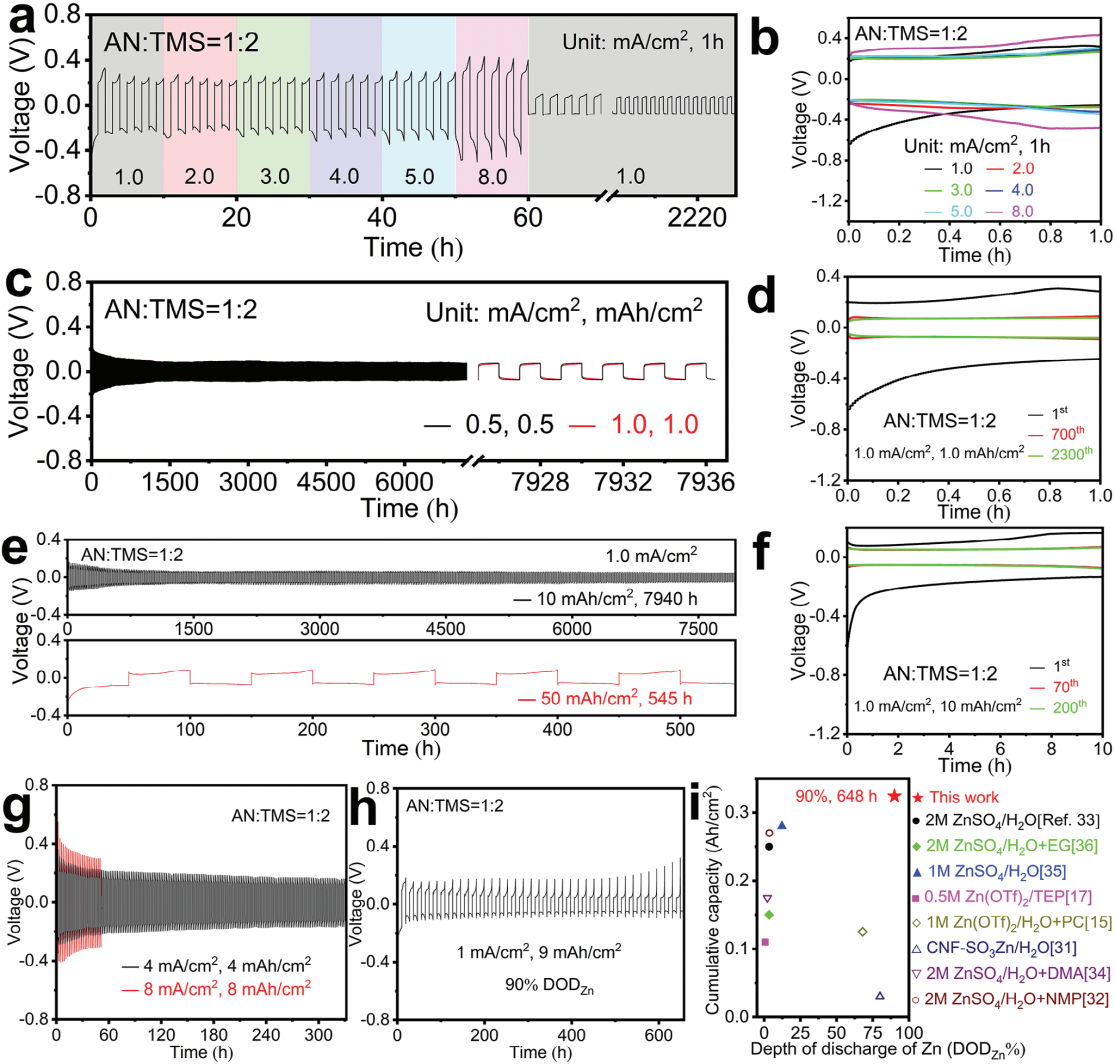
Stability and utilization of the Zn electrode in a 0.5 m Zn[NTf_2_]_2_/(*V*_AN_:*V*_TMS_ = 1:2) electrolyte. a,b) Rate performances at varying current densities (2 h per cycle). c–g) Galvanostatic Zn plating/stripping test in a symmetric Zn||Zn cell at diverse current and capacity densities. h) Cycling performance of a Zn||Zn/^3D^Cu cell with 90% DOD_Zn_ for the Zn/^3D^Cu electrode under 1.0 mA cm^−2^. i) The comparison of DOD_Zn_ dependence of the cumulative capacity with recent reports of other Zn electrolytes.

The impedance spectra before and after the chronoamperometry test of the Zn||Zn cell virtually coincide, and the chronoamperometry curves run almost parallel to the *x*‐axis after 600 s, both demonstrating stable Zn deposition (Figure S4a,b, Supporting Information).^[^ ^29^, ^30^ ^]^ Moreover, galvanostatic charge/discharge cycles of Zn||Zn symmetric cells were run with various current densities and capacities to investigate the rate performance of the 0.5 m Zn[NTf_2_]_2_/(*V*_AN_:*V*_TMS_ = 1:2) electrolyte with Zn electrodes. The hybrid electrolyte was found to sustain current densities up to 8.0 mA cm^−2^ (**Figure** **2**a). For current densities between 1.0 and 5.0 mA cm^−2^, the overpotentials between the charging and discharging curves are almost the same, and for 8.0 mA cm^−2^ somewhat increased, demonstrating a good rate performance of the hybrid electrolyte (Figure 2b).^[^ ^27^ ^]^ The Zn electrode showed a long‐cycle life of 7936 and 7936 h (2 h per cycle) at 0.5 and 1.0 mA cm^−2^ without signs of short circuit or degradation (Figure 2c). A Zn||Zn cell can be stably cycled for at least 7940 h at the high capacity density of 10 mAh cm^−2^ (20 h per cycle) with a depth of discharge (DOD_Zn_) of 7% for both Zn electrodes (each Zn electrode 195 mg, 141 mAh cm^−2^; Figure 2e). Even at the unusually high‐capacity density of 50 mAh cm^−2^, stable cycling over 545 h with 35% DOD_Zn_ for both Zn electrodes was possible (Figure 2e). Whether at 1.0 mA cm^−2^ and 1.0 or 10 mAh cm^−2^, the approximately coinciding charge/discharge curves of the 700th and 2300th (Figure 2d) or 70th and 200th cycles (Figure 2f), illustrate the high reversibility and stability of the Zn electrode in the hybrid electrolyte.

In high current and high capacity rapid charging/discharging processes, Zn||Zn cells were consistently cycled for 332, 120, and 52 h, respectively, at 4.0, 5.0, and 8.0 mA cm^−2^, respectively (Figure 2g and Figure S4c,d, Supporting Information). In accordance with the reported methodology,^[^ ^31^ ^]^ we also performed a high utilization test with a Zn||Zn/^3D^Cu cell (^3D^Cu is copper foam) over 648 h at 90% DOD_Zn_ for the Zn/^3D^Cu electrode (Figure 2h). The cumulative capacity of 0.32 Ah cm^−2^ at 90% DOD_Zn_ is superior among a series of latest published results (Figure 2i).^[^ ^15^, ^17^, ^31^, ^32^, ^33^, ^34^, ^35^, ^36^ ^]^

### 2.2 The Zn Plating/Stripping Mechanism

**Figure 3:**
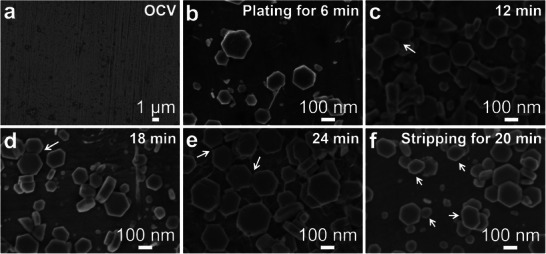
The Zn plating/stripping mechanism in the 0.5 m Zn[NTf_2_]_2_/(*V*_AN_ : *V*_TMS_ = 1:2) electrolyte investigated in a Zn||SS cell at 1.0 mA cm^−2^ by SEM images. a–e) The Zn plating morphology on the SS foil. a) The pristine SS foil at open circuit voltage state and Zn plating for b) 6 min, c) 12 min, d) 18 min, and e) 24 min. f) Plating for 30 min firstly, then stripping for 20 min.

Motivated by the high CE (Figure 1) and long cycle life and high Zn utilization (Figure 2), we can convincingly conclude that the 0.5 m Zn[NTf_2_]_2_/(*V*_AN_:*V*_TMS_ = 1:2) hybrid electrolyte plays an essential role in promoting and stabilizing the electrochemistry of Zn. To gain a deeper insight into the electrode processes, we took a series of SEM images of Zn electrodes after various times of plating or stripping at 1.0 mA cm^−2^ (**Figure** **3**). As the plating time increases, the number of hexagonal Zn platelets increases. While the diameter of the individual crystals appears to be limited to about 200–250 nm, their thickness reaches 60–90 nm (Figure 3a–e). The majority of the platelets is oriented parallel to the substrate, so that their hexagonal (001) faces are parallel. This allows for epitaxial overgrowth by other crystallites. The strongly preferred orientation is also seen in the diffraction pattern of the deposited layer, which only shows the 002 reflection of Zn (Figure S5, Supporting Information). The growth mechanism is thus a combination of layer and island growth (Stranski–Krastanov mechanism), but with a preference for layer‐by‐layer growth leading to hexagonal prisms with moderate aspect ratios. In this way, the formation of dendrites is prevented and an overall flat Zn deposit results. During stripping, the edges of the hexagons are gradually rounded, indicating an edge‐dissolution mechanism (Figure 3f).^[^ ^37^ ^]^ This is consistent with calculations that assign the lowest surface energy to the (001) face and almost three times the energy to the (111) and (101) faces.^[^ ^38^ ^]^

A Zn electrode after 26 cycles at 8.0 mA cm^−2^ showed a flat but not dense nanostructured morphology consisting of rounded platelets (Figure S6a–c, Supporting Information). Their preferred orientation increased the ratio of the X‐ray diffraction intensities *I*_002_ : *I*_101_ to 0.36 compared to 0.08 for statistical orientation (Figure S6d, Supporting Information).

### 2.3 Solvation Structure

**Figure 4:**
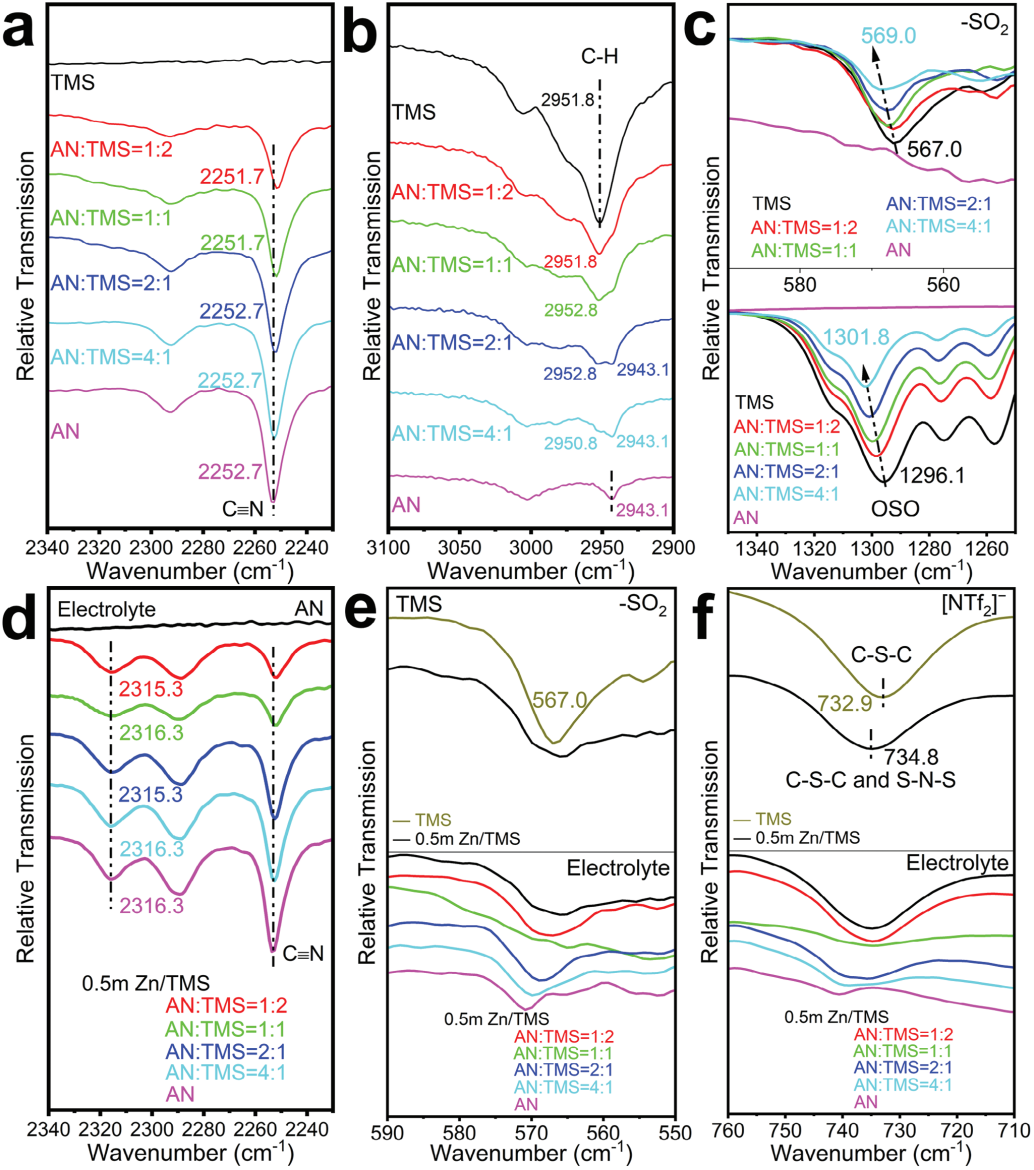
a–c) FT‐IR spectra of pure TMS and AN as well as of their mixtures. d–f) FT‐IR spectra of 0.5 m Zn[NTf_2_]_2_/(*V*_AN_:*V*_TMS_ = 0:1, 1:2, 1:1, 2:1, 4:1, and 1:0) electrolytes in selected ranges.

Vibrational FT‐IR spectroscopy and quantum‐chemical methods were used to investigate the Zn^2+^ solvation sheath structure, which is most possibly the reason for the Zn plating/stripping morphology.^[^ ^39^ ^]^ To better understand the pseudo‐ternary system of AN, TMS, and Zn[NTf_2_]_2_ that constitutes the hybrid electrolyte, we first examined the mixture of AN and TMS spectroscopically. As the content of TMS gradually increases, the C≡N stretching vibration (υ_2_‐mode) slightly shifts from 2252.7 to 2251.7 cm^−1^ (**Figure** **4**a) and the C─C vibration frequency in AN (Figure S7a, Supporting Information) shifts from 918.0 to 917.1 cm^−1^,^[^ ^40^ ^]^ which hints at weak C≡N···H hydrogen bonds between AN and TMS.^[^ ^40^, ^41^ ^]^ In the opposite direction, when AN is added to TMS (Figure 4b), the C─H vibrations of TMS slightly shift from 2951.8 to 2950.8 cm^−1^,^[^ ^42^ ^]^ the wagging vibration of the ─SO_2_ group shift from 567.0 to 569.0 cm^−1^ (upper part of Figure 4c),^[^ ^43^ ^]^ and the antisymmetric O─S─O stretching mode from 1296.1 to 1301.8 cm^−1^ (lower part of Figure 4c),^[^ ^44^ ^]^ all of which supports the assumption of weak hydrogen bonds.

The dissolution of Zn[NTf_2_]_2_ changes the structure of the complex network of hydrogen bonds in the solvent. A new absorption band appears at 2316 cm^−1^, which most probably originates from AN molecules that interact with ions of the dissolved salt (pink line in Figure 4d vs Figure 4a).^[^ ^40^ ^]^ Moreover, the –SO_2_ vibration, which is found at 567.0 cm^−1^ for the pure TMS, is broadened when the salt is added (upper part of Figure 4e). In the same wavenumber range, at about 570 cm^–1^, an absorption indicates interactions between AN molecules and ions of the dissolved salt (lower part of Figure 4e and upper part of Figure 4c).

[NTf_2_]^−^ has a characteristic asymmetric stretching frequency *v*_as_(SNS), which is found at about 741.6 cm^−1^ in the case of crystalline Zn[NTf_2_]_2_.^[^ ^45^ ^]^ This vibration mode is regarded to be rather susceptible to the change of the environment. However, the antisymmetric C─S─C stretching vibration of TMS at 732.9 cm^−1^ (for pure TMS)^[^ ^44^ ^]^ leads to an overlap (C─S─C and S─N─S) and a common band at 734.8 cm^−1^ (upper and lower part of Figure 4f).

**Figure 5:**
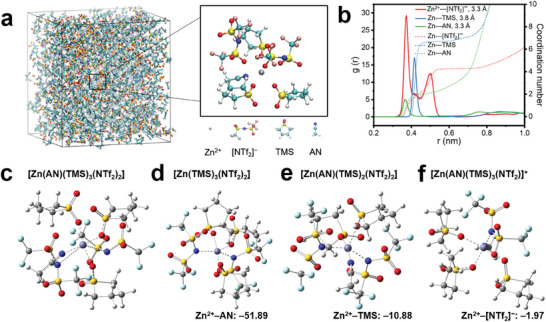
MD simulations of the Zn^2+^ solvation sheath structures in 0.5 m Zn[NTf_2_]_2_/(*V*_AN_:*V*_TMS_ = 1:2) electrolyte. a) A snapshot of the Zn^2+^ solvent box structure after MD simulations and b) the corresponding RDF. Results of the DFT geometry optimization of c) the [Zn(AN)(TMS)_3_(NTf_2_)_2_] complexes and partly stripped complexes d) [Zn(TMS)_3_(NTf_2_)_2_], e) [Zn(AN)(TMS)_2_(NTf_2_)_2_], and f) [Zn(AN)(TMS)_3_(NTf_2_)]^+^ with corresponding binding energies (kJ mol^−1^).

Molecular dynamics (MD) simulations^[^ ^46^ ^]^ were performed to learn more about the Zn^2+^ solvation in the 0.5 m Zn[NTf_2_]_2_/(*V*_AN_ : *V*_TMS_ = 1:2) electrolyte (**Figure** **5**a). The MD simulations confirm that AN, TMS and [NTf_2_]^−^ are all adjacent to the Zn^2+^ cation. Within a sphere of 0.4 nm, the average coordination number is 1.86 for AN, 3.54 for TMS, and 1.77 for [NTf_2_]^−^. Remarkably, the radial distribution function (RDF) starts at 3.3 Å for Zn^2+^–AN and Zn^2+^–[NTf_2_]^−^ and at 3.8 Å for Zn^2+^–TMS (Figure 5b). These distances are longer than for a conventional coordination. In aqueous systems, the nearest atoms to Zn^2+^ have been reported at distances between 2 Å^[^ ^14^, ^15^, ^47^, ^48^ ^]^ and 3.1 Å.^[^ ^49^ ^]^ The Zn^2+^ cation in the hybrid electrolyte can thus be considered to be weakly solvated.

Nyquist impedance measurements were performed to verify the presence of weakly solvated zinc cations. The uncycled Zn||activated carbon (AC) ion capacitor, similar to a double layer capacitor, has an impedance profile with a section consisting of an arc (high frequency, electrode ionic resistance) and a straight line (low frequency). From comparison of the data, it can be concluded that the response at the electrolyte‐electrode interface is faster in the solvent mixture *V*_AN_:*V*_TMS_ = 1:2 than in pure TMS.^[^ ^50^ ^]^ This experimental result confirms that the solvation of the zinc cation is weakened in the solvent mixture (Figure S8a, Supporting Information). When 0.5 m Zn[NTf_2_]_2_/H_2_O is used, however, the curve is very different, showing a semicircle. Using the equivalent circuit model (Figure S8b, Supporting Information), the R2 for the cycled Zn||AC ion capacitor represents the resistance to the charge transfer at the electrode/electrolyte interface, which is about 10.3 Ω for the 0.5 m Zn[NTf_2_]_2_/H_2_O and 19.2 Ω for the 0.5 m Zn[NTf_2_]_2_/(*V*_AN_:*V*_TMS_ = 1:2) electrolyte (Figure S8c,d, Supporting Information).^[^ ^27^, ^51^ ^]^

The weak interactions between AN, TMS, [NTf_2_]^−^, and Zn^2+^ may be due to the larger spatial resistance (“steric hindrance” effect) of the [NTf_2_]^−[^ ^19^ ^]^ and the interactions between the AN, TMS molecules (3.5 Å, Figure S7b, Supporting Information). However, there is no evidence for strong interactions between [NTf_2_]^−^ and AN, TMS (Figure S7c, Supporting Information). Afterwards, combining the analysis of the MD simulations and the coordination number via RDF plot, we assume there will be six ions and molecules coordinating a Zn^2+^ cation, which is in accordance with previous literature.^[^ ^52^ ^]^ The most probable complex in solution should be [Zn(AN)(TMS)_3_(NTf_2_)_2_].

Density functional theory (DFT) based calculations were performed to optimize the geometry and to determine the relative binding energy of [Zn(AN)(TMS)_3_(NTf_2_)_2_] and the partly stripped complexes with five ligands [Zn(TMS)_3_(NTf_2_)_2_], [Zn(AN)(TMS)_2_(NTf_2_)_2_] and [Zn(AN)(TMS)_3_(NTf_2_)]^+^. The aim was to get information about the reasons for the structured, dendrite‐free Zn deposits (Figure S7d‐f and Tables S1–S7, Supporting Information). As shown in Figure 5c–f, the interaction energy Zn^2+^–AN (ca. −52 kJ mol^−1^) is substantially larger than that of Zn^2+^–TMS (ca. −11 kJ mol^−1^) or Zn^2+^–[NTf_2_]^−^ (ca. −2 kJ mol^−1^). Hence, we proposed a solvents‐induced model for the textured Zn deposition, in which the [NTf_2_]^−^ anions are first stripped off (Figure 5f vs Figure 5d,e and Figure S7g, Supporting Information).^[^ ^53^ ^]^ This leaves positive charges on the complex and increases the attractive interaction with the negatively charged cathode. Although the Zn^2+^–AN is stronger than the Zn^2+^–TMS interaction, the larger number of TMS molecules can lead to a competition in the subsequent stripping of ligands.

It is known that the oxygen atom of TMS strongly interacts with stainless steel or zinc electrodes, which can stabilize [Zn(AN)(TMS)_3_]^2+^ on the substrate.^[^ ^52^, ^53^, ^54^ ^]^ It has also been shown that organic solvent molecules with oxygen atoms (e.g., 1,2‐dimethoxyethane, DME)^[^ ^42^ ^]^ strongly adsorb on the Zn(001) surface, thereby blocking it. The (partially stripped) Zn^2+^ complexes then preferable bind to side facets, such as Zn(111) or Zn(101), which favors lateral growth and thus a flat morphology. Thus, the weak solvation of the Zn^2+^ cation rationalizes transport efficiency in the electrolyte, reversibility of electrode processes and, combined with specific solvent molecules, the flat morphology of the deposited Zn.

### 2.4 Performance of a Zn||Activated‐Carbon Ion Hybrid Capacitor

**Figure 6:**
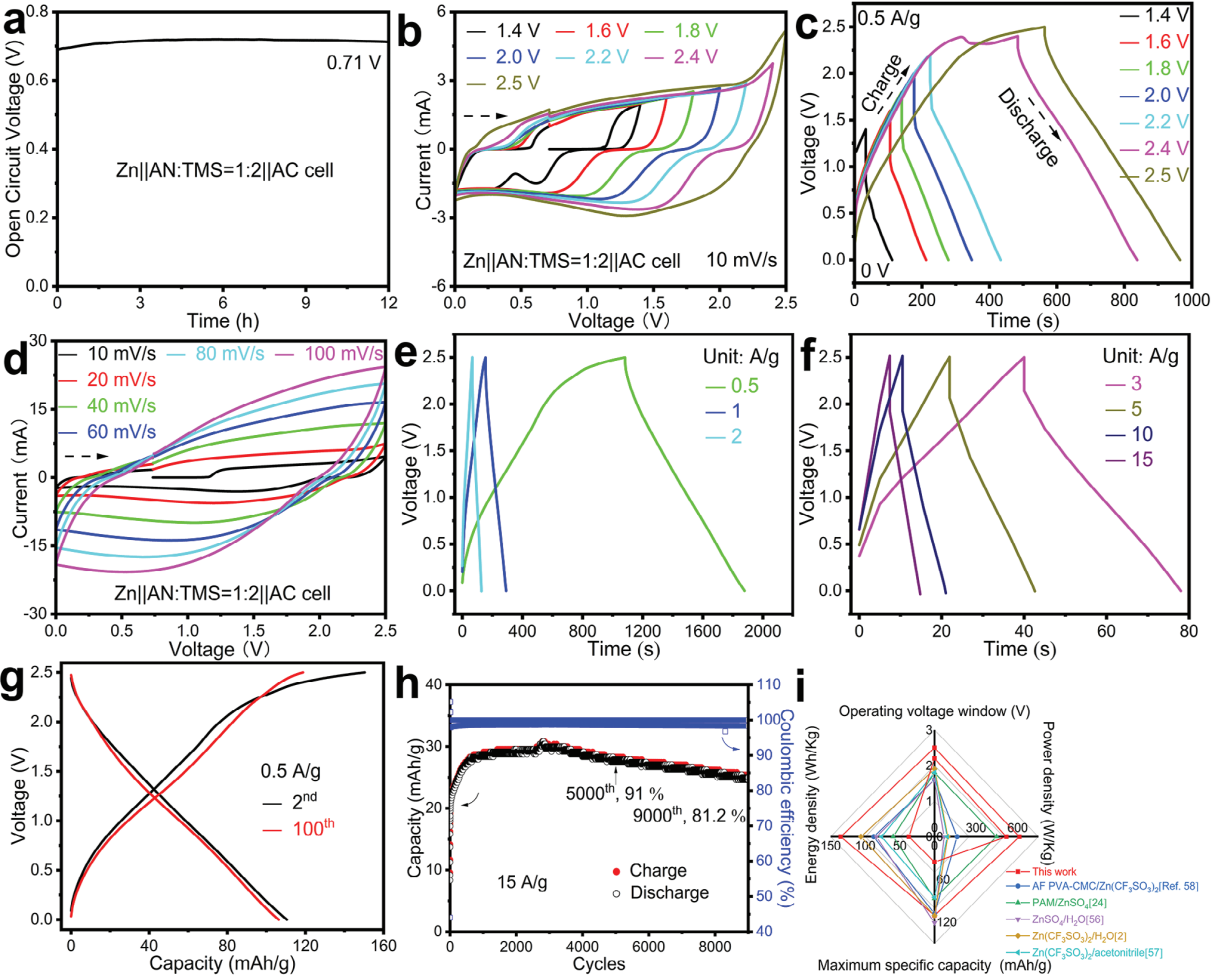
Electrochemical performance of a Zn||activated‐carbon ion hybrid capacitor employing 0.5 m Zn[NTf_2_]_2_/(*V*_AN_ : *V*_TMS_ = 1:2) electrolyte. a) The stability curve of the open circuit voltage within 12 h rest. b) CVs with stepwise increase in positive cell voltage limits from 1.4 to 2.5 V. c) Charge/discharge profiles with different positive cell voltage limits (discharge to 0 V). d) CVs between 0 and 2.5 V at different scan rate. e,f) Rate performance between 0 and 2.5 V. g) Selected charge/discharge profiles at 0.5 A g^−1^ and h) long‐term cycling at 15 A g^−1^. i) Comparison (radar plot) of the Zn||activated‐carbon ion hybrid capacitor (based on data operating between 0–2.2 and 0–2.5 V) with other recently reported devices.

The performance of the 0.5 m Zn[NTf_2_]_2_/(*V*_AN_:*V*_TMS_ = 1:2) electrolyte was evaluated in a Zn ion hybrid capacitor with activated carbon (AC) as counter electrode. After 12 h rest, the open circuit voltage of the capacitor had stabilized at 0.71 V (**Figure** **6**a). The operating voltage was first investigated by CVs in different voltage ranges (Figure 6b). As the voltage increased at a constant rate of 10 mV s^−1^, a quick upturn in current to about 1.5 mA was observed, followed by an only slightly increasing, almost constant current. Above 2.2 V, the regime changes as the current again increases strongly. The same charging process monitored over time at a constant current of 0.5 A g^−1^ (Figure 6c and Figure S9, Supporting Information), showed an instant jump of voltage followed by an almost linear increase, which could be explained by a surface‐near process and a diffusion‐controlled process. Above 2.2 V, the charging process slows down considerably. This can be caused either by a changed kinetics or by irreversible processes, such as the decomposition of the electrolyte or oxidation of the carbon electrode.^[^ ^55^ ^]^ The discharge process under the same conditions proceeds very quickly in the early stage, but then slows down to a linear decrease of voltage. The change between both regimes is quite well defined, and the initial voltage drop of about 0.5 V is essentially independent of the maximum charge (surface capacitance) and possibly due to equivalent resistance of the cell. Higher scan rates gradually shift the CV curves to higher currents (Figure 6d), which is due to diffusion limitations of the ions.^[^ ^55^ ^]^ None of the CVs shows obvious redox peaks, suggesting that the device is a classical double layer capacitor. Higher current densities in the time‐dependent measurements accelerate the charge and discharge processes but do not change them fundamentally (Figure 6e,f).

At 0.5 and 1.0 A g^−1^ between 0 and 2.0 V, the coulombic efficiency of the Zn||AC ion hybrid capacitor was about 99.4% after 93 cycles (Figures S10a,b, Supporting Information) or 99.7% (Figure S10c,d, Supporting Information) after 224 cycles in a second experiment. At 0.5 A g^−1^ between 0 and 2.2 V, the coulombic efficiency was about 98.2% (Figures S10e,f, Supporting Information) after 84 cycles. A coin cell with 0.48 mg (0.42 mg cm^−2^) of activated carbon operated at a maximum voltage of 2.1 V exhibits a specific discharge capacity of up to 35.7 mAh g^−1^ at 0.5 A g^−1^ (Figure S10e, Supporting Information) with a specific capacitance of ≈62.1 F g^−1^, an energy density of about 37 Wh kg^−1^ at a power density of 519 W kg^−1^. The calculation is based on the discharge capacity of the activated carbon. Increasing the mass of AC to 13.95 mg (12.3 mg cm^−2^), results in a similar discharge capacity (31.5 mAh g^−1^ at 0.1 A g^−1^, Figure S10j, Supporting Information), indicating hindered transport inside the AC.

Despite the presence of side reactions, coin cells were stably cycled also at 0.5 A g^−1^ at the increased voltage of 2.5 V. The specific discharge capacity was 110 mAh g^−1^ (Figure 6g) with a specific capacitance of about 162 F g^−1^, an energy density of about 135 Wh kg^−1^ and a power density of 613 W kg^−1^. The calculation is based on the discharge capacity of activated carbon with a mass of 0.36 mg (0.32 mg cm^−2^). The 2nd and 100th charge and discharge curves of the capacitor mostly overlap with each other (Figure 6g), also demonstrating the reversibility of the processes. While higher specific capacity can be achieved when operating at low currents, cycle life may also be limited due to electrolyte decomposition at this high voltage (Figure 6g). At the high current density of 10 A g^−1^ applying up to 2.5 V, the device delivers a capacity retention of 95% after 6000 cycles (Figure S10g, Supporting Information). At the even higher current density of 15 A g^−1^, during the initial 600 cycles (14 s each), the specific capacity increases, indicating an activation process of the carbon electrode making more active material accessible. The capacity retention is 91% after 5000 cycles, 81% after 9000 cycles (Figure 6h), and 79% after 10 000 cycles (Figure S10h,i, Supporting Information). The specific capacitance is about 58 F g^−1^, the energy density is 29.3 Wh kg^−1^ and the power density is 14 50 W kg^−1^. The calculation is based on the discharge capacity of activated carbon with a mass of 0.54 mg (0.48 mg cm^−2^) at a maximum voltage of 1.9 V.

The Zn||Zn cell, the Zn||SS cell, and the Zn||AC ion capacitor using 0.5 m Zn[NTf_2_]_2_/(*V*_AN_:*V*_TMS_ = 1:2) all outperform corresponding cells with a 0.5 m Zn[NTf_2_]_2_/H_2_O electrolyte that were measured for comparison (Figure S11, Supporting Information). The combination of maximum operating voltage, specific capacity, energy density and power density considerably exceeds the performance of several recently reported devices with various Zn electrolytes, such as aqueous, organic and solid state electrolytes (Figure 6i).^[^ ^2^, ^24^, ^56^, ^57^, ^58^ ^]^

## 3 Conclusion

To summarize, we have developed an electrolyte that solves the major problems inhibiting the use of zinc ion capacitors. The special combination of acetonitrile and tetramethylene sulfone as solvents and [NTf_2_]^–^ as anion of the zinc salt creates an essentially non‐coordinating liquid environment for the Zn^2+^ cations. This is the key for fast transport in the solution, highly reversible electrode processes and a flat morphology of the deposited zinc layer. The oxidation stable organic electrolyte avoids the parasitic reactions of water‐based electrolytes and the associated generation of gas. In a simply constructed Zn||activated‐carbon ion hybrid capacitor, the advantageous properties of the electrolyte allow an operating voltage of 2.0–2.5 V and provide excellent cycling stability also at a high depth of discharge. Very high current densities can be applied enabling fast charging and discharging over a large number of cycles. One of the possible applications of such a zinc ion capacitor could be the recuperation of kinetic energy in automobiles. Furthermore, the chemical concept pursued here should be transferable to other systems.

## 4 Experimental Section

### Chemicals

Zn foil (≈250 µm) was purchased from Alfa Aesar, Zn[NTf_2_]_2_ (99.5%) from Solvionic, tetramethylene sulfone (99%) from Merck Schuchardt, acetonitrile from Sigma‐Aldrich (anhydrous, 99.8%) and Fisher Chemical (HPLC gradient grade, ≥99.9%). Activated carbon (AC) powder was obtained from Sigma‐Aldrich and Whatman glassy fiber (GF) from cytiva (thickness in fluffy state about 0.5 mm). Demineralized water, Zn acetate (Zn(OAc)_2_, Zn(CH_3_COO)_2_), Zn trifluoromethanesulfonate (Zn[OTf]_2_, Zn(CF_3_SO_3_)_2_), polytetrafluoroethylene (PTFE, 10 wt.% dispersion in H_2_O), 2‐propanol, stainless steel foil (304, 25 µm thickness) and stainless‐steel mesh were laboratory grade. Guangdong Canrd New Energy Technology Co., Ltd. supplied coin cell shells (CR2032) with spring and stainless‐steel spacer (1.0 mm thickness) and three‐dimensional Cu foam (^3D^Cu, 0.3 × 200 × 300 mm^3^, 35.2 g).

### Preparation of Electrolytes

AN and TMS were mixed in defined volumetric ratios (*V*_AN_ : *V*_TMS_ = 1:4, 1:2, 1:1, 2:1, and 4:1), then Zn[NTf_2_]_2_ salt was added to form 0.5 mol L^−1^ (0.5 m) transparent and stable solutions after stirring for 8 h. For comparison, 0.5 m solutions of Zn[NTf_2_]_2_ in pure TMS, pure AN and pure H_2_O was also prepared. Additionally, 0.5 m solutions of Zn(OAc)_2_ and Zn[OTf]_2_ in AN/TMS mixtures (*V*_AN_ : *V*_TMS_ = 0:1, 1:2, 1:1, 2:1, 4:1, and 1:0) were also prepared for testing the influence of the anions on the process. Solubility test of Zn[NTf_2_]_2_ in solvents mixture (*V*_AN_:*V*_TMS_ = 1:2) at 3.0 m was also performed.

### Electrochemical Measurements

The linear sweep voltammetry (LSV), cyclic voltammogram (CV), and open circuit voltage of cells were performed on VMP‐3 model of Biologic SAS controlled by EC‐LAB electrochemistry software. The electrolyte used in assembling cells was ≈150–200 µL. All cells were rested for 12 h before cycle stability test. The cycle stability of cells was tested using Neware BTS4000‐5 V 10 mA Battery Testing System (Xiamen AOT Electronics Technology Co.) at room temperature. All samples were washed with AN and ethanol, then dried in air and stored in an argon glovebox before further characterization. For cycled Zn electrodes, in addition to cleaning step described above, ultrasonic cleaning in ethanol for 10–15 min was used to remove as much of the GF separator residue as possible.

The electrochemical stability window of the electrolyte was estimated via LSV through the first cycle. Zn foil, punched and pressed into 12 mm diameter discs, was assembled in the glovebox into Zn||SS spacer coin‐shell (type 2032) cells (Manual Coin Cell Crimper AOT‐HCM‐20, encapsulation pressure about 50 kg cm^−2^) with GF separator. The test was conducted in a potential window from 0 V to ≈6 V (vs Zn/Zn^2+^) with a sweep rate of 2 mV s^−1^. Zn||SS (spacer, 1.0 mm thickness) and Zn||SS (foil, 25 µm thickness) coin‐type cells using different electrolytes and GF separator for both were also assembled to test CE and the morphology of Zn deposit, separately. For the CE test, Zn||SS cells were cycled for 1 h at 1.0 mA cm^−2^ for the discharging process and a cut‐off potential of 0.5 V at 1.0 mA cm^−2^ for the charging process. In addition, symmetric Zn||Zn coin‐type cells with 0.5 m Zn[NTf_2_]_2_/(*V*_AN_ : *V*_TMS_ = 1:4, 1:2) or 0.5 m Zn[NTf_2_]_2_/H_2_O and double‐layered GF separator were assembled to test cycling stability at different currents and operating durations. The Zn||^3D^Cu cell was first assembled by using 0.5 m Zn[NTf_2_]_2_/(*V*_AN_ : *V*_TMS_ = 1:2) and double‐layered GF separator, and then Zn was deposited on^3D^Cu for 10 h under 1.0 mA cm^−2^. Then, the cycling performance of a Zn||Zn/^3D^Cu cell with 90% depth of discharge (DOD_Zn_) for the Zn/^3D^Cu electrode was performed under 1.0 mA cm^−2^ for 9 h. The Zn||^3D^Cu cell was first assembled, and then zinc was deposited on ^3D^Cu for 10 h at 1.0 mA cm^−2^. The Zn/^3D^Cu electrode was obtained by unpacking the cell. Then, the cycling performance of Zn/^3D^Cu||SS cells with 50% DOD_Zn_ was performed. The impedance spectra before and after chronoamperometry measurements were determined in the frequency range of 100 mHz to 1 MHz at a perturbation voltage of 10 mV. The chronoamperometry profile of Zn||Zn symmetrical cell was tested under a polarization voltage of 50 mV in 0.5 m Zn[NTf_2_]_2_/(*V*_AN_:*V*_TMS_ = 1:2) electrolyte. The impedance spectrum was fitted using Zfit of Biologic.

Active carbon (AC) electrodes were prepared using AC as the active material, polytetrafluoroethylene as binder (*m*_AC_/*m*_PTFE_ = 90 mg : 10 mg) and about 1 mL of 2‐propanol. After stirring overnight, the suspension was poured as a thin film onto a heating table to evaporate the solvent. The jelly‐like film was rolled on a stainless‐steel mesh with 12 mm diameter. All electrodes were dried in an oven at 90 °C overnight. The mass loading of the AC was between 0.24 and 12.3 mg cm^−2^ (calculated from the area of stainless steel mesh). Furthermore, Zn foil, AC electrode, ≈150–200 µL of 0.5 m Zn[NTf_2_]_2_/(*V*_AN_:*V*_TMS_ = 1:2) electrolyte or 0.5 m Zn[NTf_2_]_2_/H_2_O and glassy fiber separator was applied to assemble the Zn||AC capacitor. The specific capacity and current density were based on activated carbon. Afterwards, Zn||AC ion capacitor was cycled at different conditions. The specific gravimetric capacitance was calculated based on the equation: *C* = *It*/(*mU*), where *I* was the charge/discharge current, *t* the charge/discharge time, *m* the mass of the activated carbon, and *U* the voltage difference of the discharge operation curve. The energy density was calculated by *E* = *CV* ^2^/2, where *C* is the specific gravimetric capacitance calculated above, and *V* is the operation voltage. The power density was calculated by the equation: *P* = *E*/*t*, where *E* is the energy density and *t* the corresponding discharge time.^[^ ^2^ ^]^

### Powder X‐ray Diffraction (PXRD)

Powder X‐ray Diffraction was carried out on an Empyrean diffractometer (PAN‐analytical) at 296(1) K equipped with a curved Ge(111) monochromator in Bragg‐Brentano geometry using Cu‐Kα_1_ radiation (*λ* = 154.0598 pm).

### Infrared (IR) Measurement

About 0.3 mL of each sample were dripped on a sample table for the IR measurements. Vibrational spectra were measured with a Bruker Vertex 70 FTIR spectrometer with attenuated total reflection (ATR) accessory in a radiation range from 400 to 4000 cm^–1^. Data analysis was performed with the program OPUS.

### Scanning Electron Microscopy (SEM) Analysis

The samples were stuck to carbon adhesive (laboratory grade) and this was glued on a sample holder. SEM images were taken with a Hitachi SU8020 microscope.

### Molecular Dynamics Simulations

All molecules were optimized using Gaussian 16 with the b3lyp^[^ ^59^ ^]^/def2tzvp^[^ ^60^ ^]^ level of theory. Afterwards, for the optimization, the the GAFF force field^[^ ^61^, ^62^ ^]^ was applied to both ions and small molecules via the Sobtop web server.^[^ ^46^ ^]^ The Packmol^[^ ^63^ ^]^ program was used to model the input file for molecular dynamics simulations, and the solute box was set to 5 × 5 × 5 nm^3^. In the solvent box modeling process, Zn[NTf_2_]_2_ was added as a unit with AN and TMS molecules respectively, to the solvent box. Gromacs software version 5.1.3‐avx^[^ ^64^, ^65^, ^66^ ^]^ was employed for all simulation procedures. First, the energy was minimized using the steepest descent method for 50 000 steps. To pre‐equilibrate the system, the temperature was controlled at 298 K and the pressure at 1 atm, with a short‐range electrostatic cutoff of 1.2 nm, and the simulation was run for 100 ps with a time‐step of 1 fs. Then, an NPT pre‐equilibration was performed, wherein the temperature was lowered from 298 to 150 K for 100 ps, held at 150 K for 100 ps, and then increased back to 298 K for 100 ps. Finally, the equilibrated state of the solvent box at 298 K was generated for another 100 ps. Subsequently, under the equilibrium parameter that employed Nose‐Hoover thermostat T‐coupling and Parrinello‐Rahman P‐coupling, the final molecular dynamics simulation was run for 1.5 ns with a time constant of 2 fs, to explore the coordination situation inside the hybrid solution by analyzing the radial distribution function plot. The coordination number was calculated using Python. All the computational calculation including are processed by Gaussian 16 program package.^[^ ^60^ ^]^ Considering the correction potential of long‐range behavior, the structure‐optimization is processing on pbe0^[^ ^67^ ^]^/def2svp^[^ ^68^, ^69^ ^]^ level, meanwhile the dispersion correction item BJ‐damping DFT‐D3(BJ)^[^ ^70^, ^71^, ^72^ ^]^ was supplemented. Once the complex electron density of transition metal Zn is taken into account, the structure optimizations were carried out on pbe0^[^ ^67^ ^]^/def2qzvp^[^ ^68^, ^69^ ^]^ level. The binding energy is calculated by the following,

(1) $$\begin{array}{ccc} E_{\mathrm{binding}}\left(Zn^{2+}-\mathrm{AN}\right) & = & E\left(\left[\mathrm{Zn}\left(\mathrm{AN}\right){\left(\mathrm{TMS}\right)}_{3}{\left(\mathrm{NT}f_{2}\right)}_{2}\right]\right)\\ & & -\;E\left(\left[\mathrm{Zn}{\left(\mathrm{TMS}\right)}_{3}{\left(\mathrm{NT}f_{2}\right)}_{2}\right]\right)-E\left(\mathrm{AN}\right) \end{array}$$

(2) $$\begin{array}{ccc} E_{\mathrm{binding}}\left(Zn^{2+}-\mathrm{TMS}\right) & = & E\left(\left[\mathrm{Zn}\left(\mathrm{AN}\right){\left(\mathrm{TMS}\right)}_{3}{\left(\mathrm{NT}f_{2}\right)}_{2}\right]\right)\\ & & -\;E\left(\left[\mathrm{Zn}\left(\mathrm{AN}\right){\left(\mathrm{TMS}\right)}_{2}{\left(\mathrm{NT}f_{2}\right)}_{2}\right]\right)-E\left(\mathrm{TMS}\right) \end{array}$$

(3) $$\begin{array}{ccc} E_{\mathrm{binding}}\left(Zn^{2+}-{\left[\mathrm{NT}f_{2}\right]}^{-}\right) & = & E\left(\left[\mathrm{Zn}\left(\mathrm{AN}\right){\left(\mathrm{TMS}\right)}_{3}{\left(\mathrm{NT}f_{2}\right)}_{2}\right]\right)\\ & & -\;E\left({\left[\mathrm{Zn}\left(\mathrm{AN}\right){\left(\mathrm{TMS}\right)}_{3}\left(\mathrm{NT}f_{2}\right)\right]}^{+}\right)\\ & & -\;E\left({\left[\mathrm{NT}f_{2}\right]}^{-}\right) \end{array}$$

## Conflict of Interest

The authors declare no conflict of interest.

## Supporting information

Supporting Information

## Data Availability

The data that support the findings of this study are available in the supplementary material of this article.
