# Supplementary material for: Key to High Performance Ion Hybrid Capacitor: Weakly Solvated Zinc Cations
Source: Adv Sci (Weinh). 2023 Nov 23;11(3):2305532. doi: 10.1002/advs.202305532 (PMC10797483; doi:10.1002/advs.202305532)
Supplement: Supplementary file 1 — Supporting Information [file ADVS-11-2305532-s001.pdf]

ADVANCED SCIENCE
Open Access

Supporting Information

for *Adv. Sci.*, DOI 10.1002/advs.202305532

Key to High Performance Ion Hybrid Capacitor: Weakly Solvated Zinc Cations

*Peng Chen, Xiaohan Sun, Bernd Plietker and Michael Ruck**

Supporting Information

Key to High Performance Ion Hybrid Capacitor: Weakly Solvated Zinc Cations

*Peng Chen, Xiaohan Sun, Bernd Plietker, and Michael Ruck**

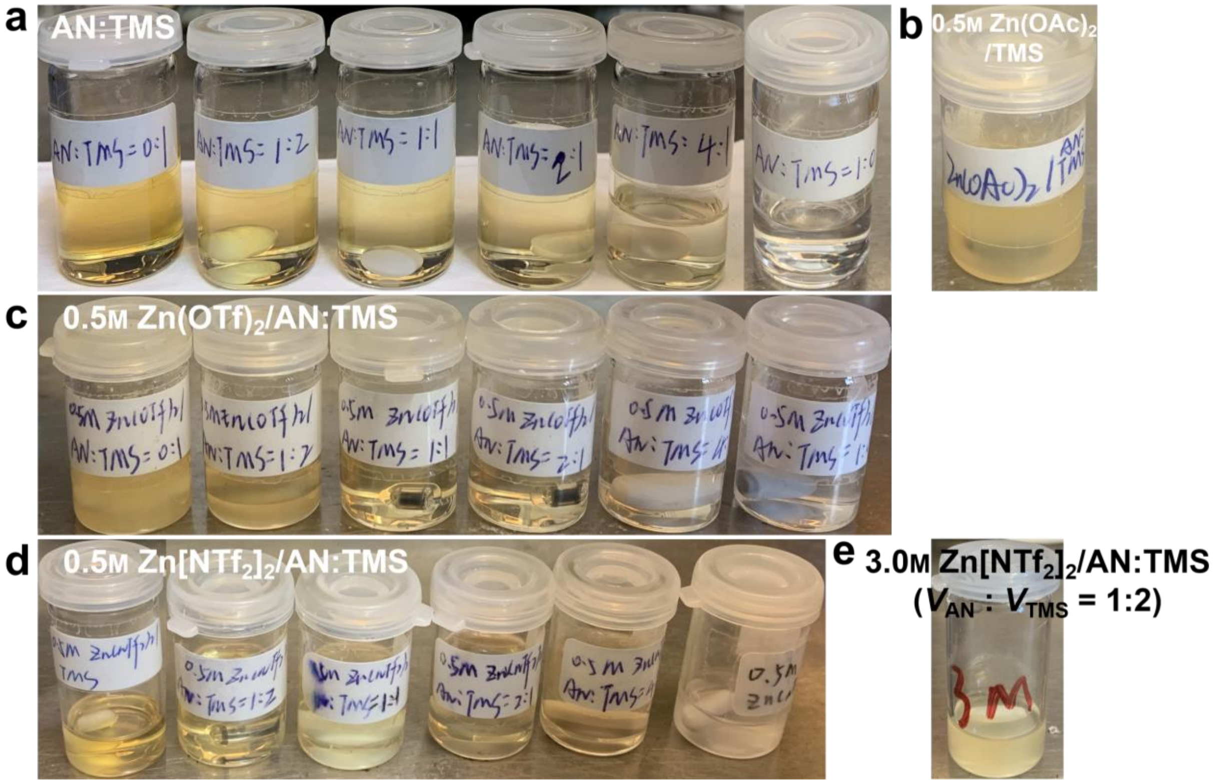

Figure S1. (a) Pure TMS and AN or their mixtures by a certain volumetric ratio ($V_{AN} : V_{TMS}$ = 1:2, 1:1, 2:1 and 4:1, from left to right). **(b)** Solubility comparison of 0.5 M $Zn(OAc)_2$/TMS, **(c)** 0.5 M $Zn[OTf]_2$/($V_{AN} : V_{TMS}$ = 0:1, 1:2, 1:1, 2:1, 4:1 and 1:0, from left to right) and **(d)** 0.5 M $Zn[NTf_2]_2$/($V_{AN} : V_{TMS}$ = 0:1, 1:2, 1:1, 2:1, 4:1 and 1:0, from left to right) electrolyte. **(e)** Solubility of $Zn[NTf_2]_2$ in solvents mixture ($V_{AN} : V_{TMS}$ = 1:2) at 3.0 M.

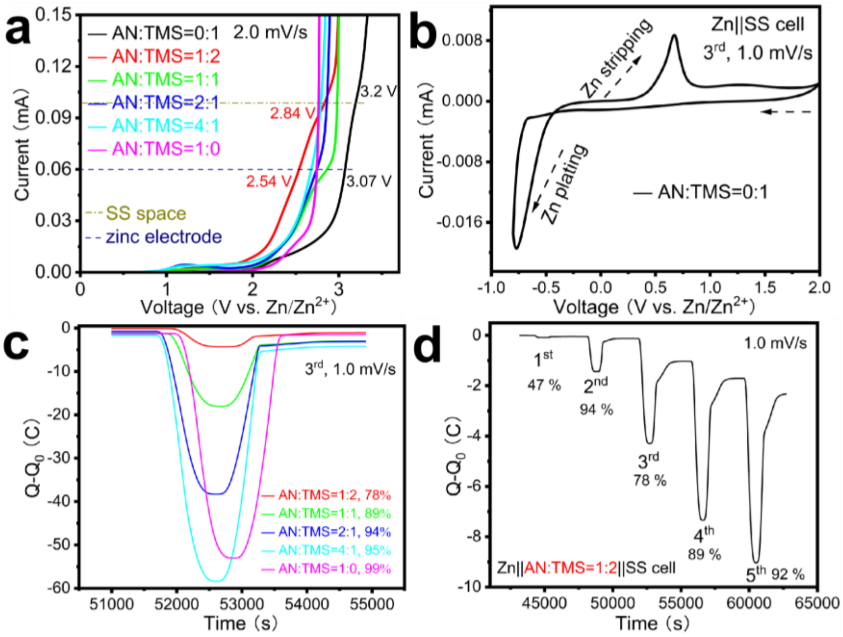

Figure S2. (a) The magnified LSV curves in Figure 1a for oxidation stability of 0.5 M $Zn[NTf_2]_2/(V_{AN}:V_{TMS}$ = 0:1, 1:2, 1:1, 2:1, 4:1 and 1:0) electrolytes in a Zn||SS cell. The dashed lines mark the decomposition potentials of SS space (diameter 16 mm) and zinc electrode (diameter 12 mm) at 0.05 mA cm^{-2}, respectively. **(b)** The CV of a Zn||SS cell with 0.5 M $Zn[NTf_2]_2$/TMS electrolyte. **(c)** Chronocoulometry curves of Zn plating/striping in 0.5 M $Zn[NTf_2]_2/(V_{AN}:V_{TMS}$ = 1:2, 1:1, 2:1, 4:1 and 1:0) electrolytes in a Zn||SS cell based on the CV curves in Figure 1b. **(d)** The first five chronocoulometry curves with 0.5 M $Zn[NTf_2]_2/(V_{AN}:V_{TMS}$ = 1:2) based on the CV curves in Figure 1c.

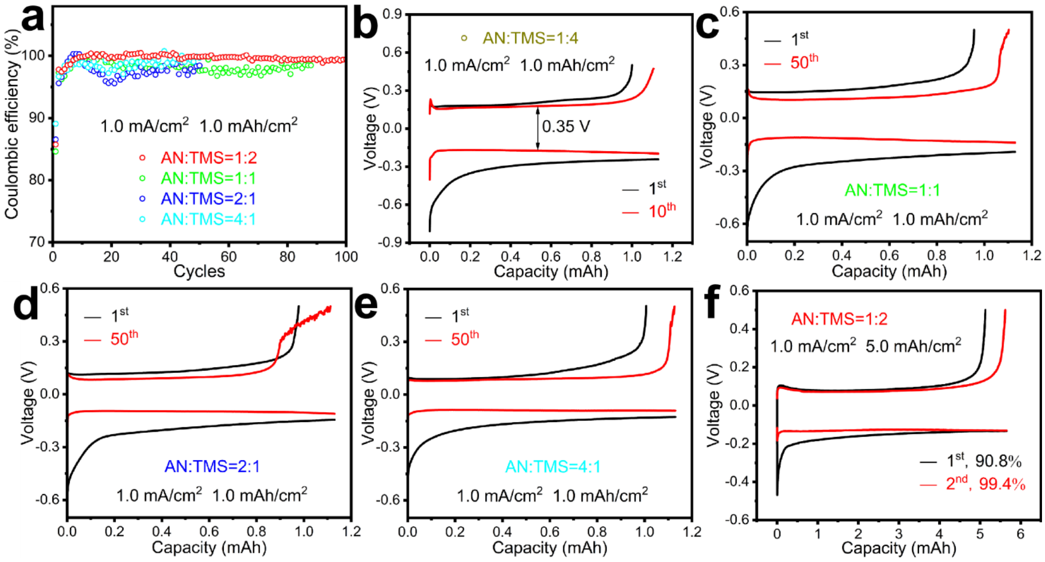

Figure S3. (a) Coulombic efficiency at 1.0 mA cm^{-2} and 1.0 mAh cm^{-2} (enlarged image of Figure 1e for first 100 cycles). Selected voltage profiles of a Zn||SS cell with **(b)** 0.5 M $Zn[NTf_2]_2/(V_{AN}:V_{TMS}$ = 1:4), **(c)** 0.5 M $Zn[NTf_2]_2/(V_{AN}:V_{TMS}$ = 1:1), **(d)** 0.5 M $Zn[NTf_2]_2/(V_{AN}:V_{TMS}$ = 2:1), and **(e)** 0.5 M $Zn[NTf_2]_2/(V_{AN} : V_{TMS}$ = 4:1) electrolyte. **(f)** 0.5 M $Zn[NTf_2]_2/(V_{AN}:V_{TMS}$ = 1:2) electrolyte at 1.0 mA cm^{-2} and 5.0 mAh cm^{-2} in a $Zn/^{3D}Cu$||SS cell with 50% DOD_{Zn} for the $Zn/^{3D}Cu$ electrode.

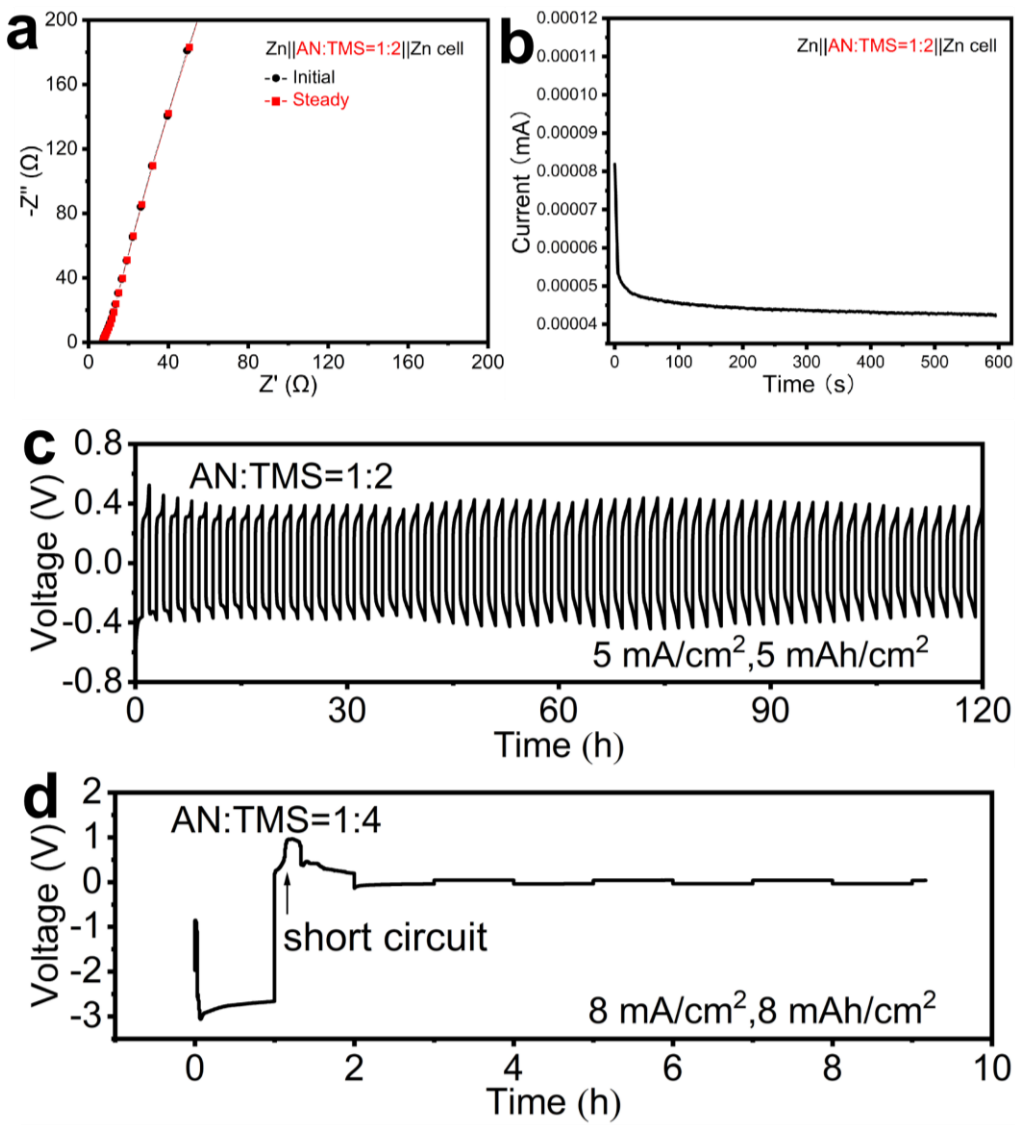

Figure S4. (a) The impedance spectra before and after chronoamperometry measurements and **(b)** the chronoamperometry profile of Zn||Zn symmetrical cell in 0.5 M Zn[NTf$_2$]$_2$/(V_{AN}:V_{TMS} = 1:2) electrolyte under a polarization voltage of 50 mV. **(c)** Cycling performance of a symmetric Zn||Zn cell in the 0.5 M Zn[NTf$_2$]$_2$/(V_{AN}:V_{TMS} = 1:2) electrolyte at 5.0 mA cm^{-2} and 5.0 mAh cm^{-2}, and **(d)** in the 0.5 M Zn[NTf$_2$]$_2$/(V_{AN}:V_{TMS} = 1:4) electrolyte at 8.0 mA cm^{-2} and 8.0 mAh cm^{-2}.

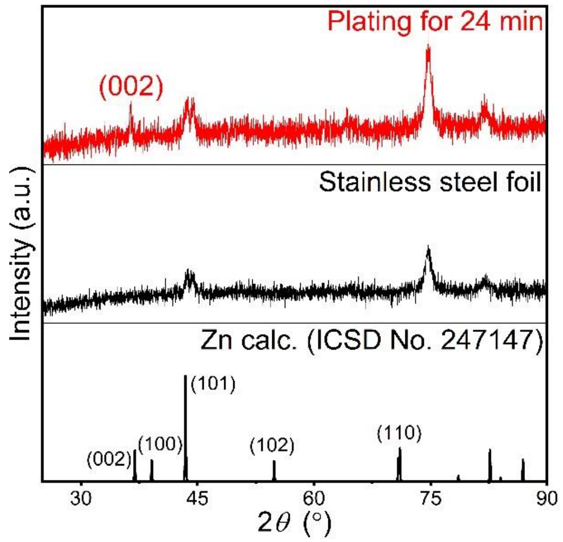

Figure S5. PXRD patterns of the pristine stainless steel foil and after Zn plating for 24 min extracted from a Zn||SS cell at 1.0 mA cm^{-2} in the 0.5 M Zn[NTf$_2$]$_2$/(V_{AN}:V_{TMS} = 1:2) electrolyte, together with its simulated reference pattern.

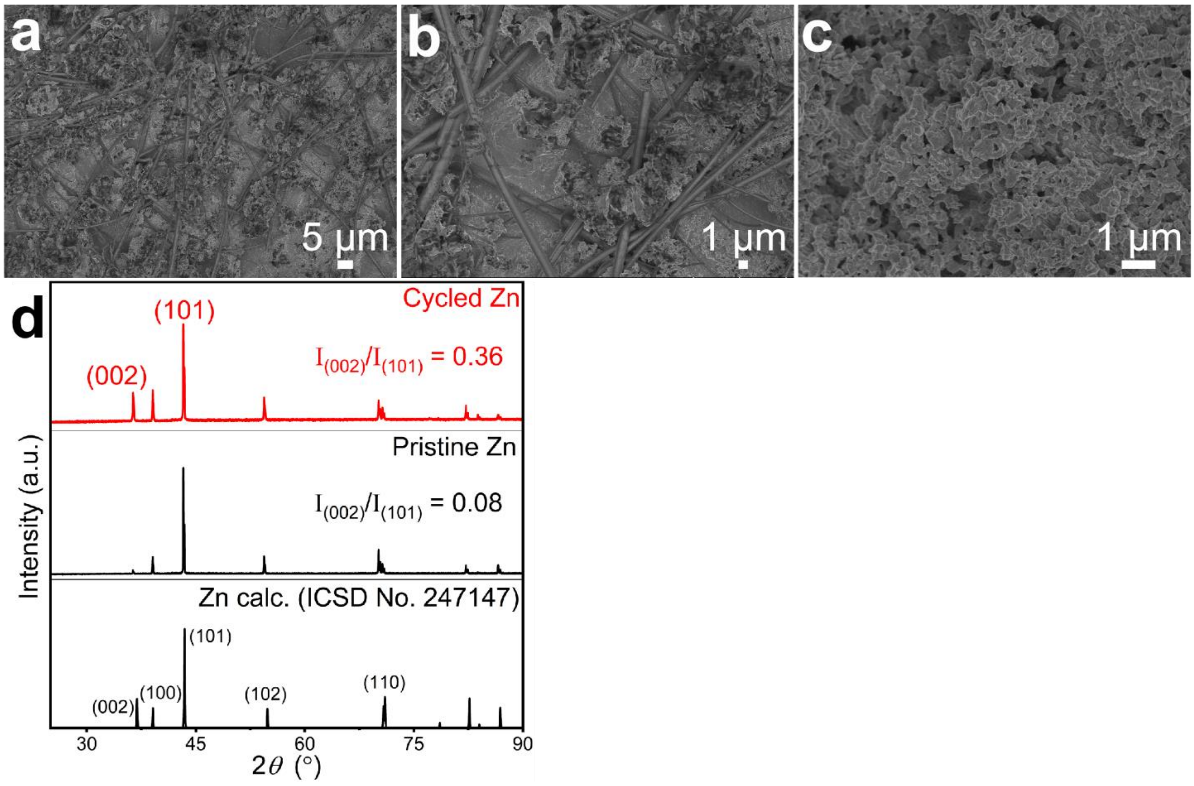

Figure S6. Characterization of zinc electrodes after 26 cycles at 8.0 mA cm^{-2} for 8.0 mAh cm^{-2} in the 0.5 M Zn[NTf$_2$]$_2$/(V_{AN}:V_{TMS} = 1:2) electrolyte. **(a-c)** SEM images (increasing magnification). **(d)** PXRD pattern.

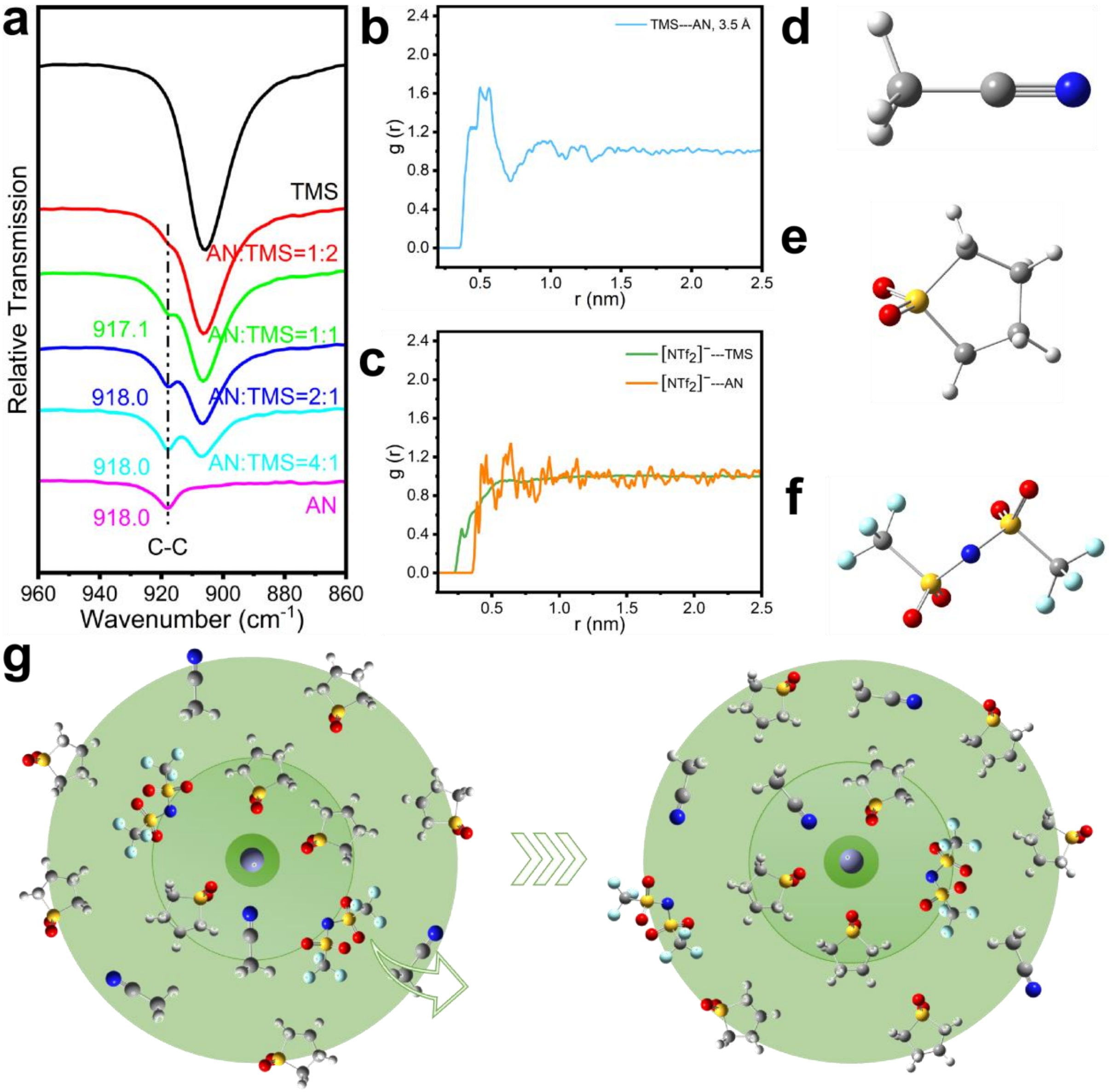

Figure S7. **(a)** Vibrational FT-IR spectra of the pure TMS and AN or their mixtures solvents in the different range. Corresponding RDF plots among **(b)** AN and TMS solvents and **(c)** solvents molecule to $[NTf_2]^-$ anion in 0.5 M $Zn[NTf_2]_2/(V_{AN}:V_{TMS} = 1:2)$ electrolyte. Results of the DFT geometry optimization of **(d)** the AN, **(e)** TMS and **(f)** $[NTf_2]^-$. **(g)** Schematic illustration of the Zn^{2+} solvation structure of $[Zn(AN)(TMS)_3(NTf_2)_2]$ complexes and after abstraction of one $[NTf_2]^-$ in the 0.5 M $Zn[NTf_2]_2/(V_{AN}:V_{TMS} = 1:2)$ electrolyte.

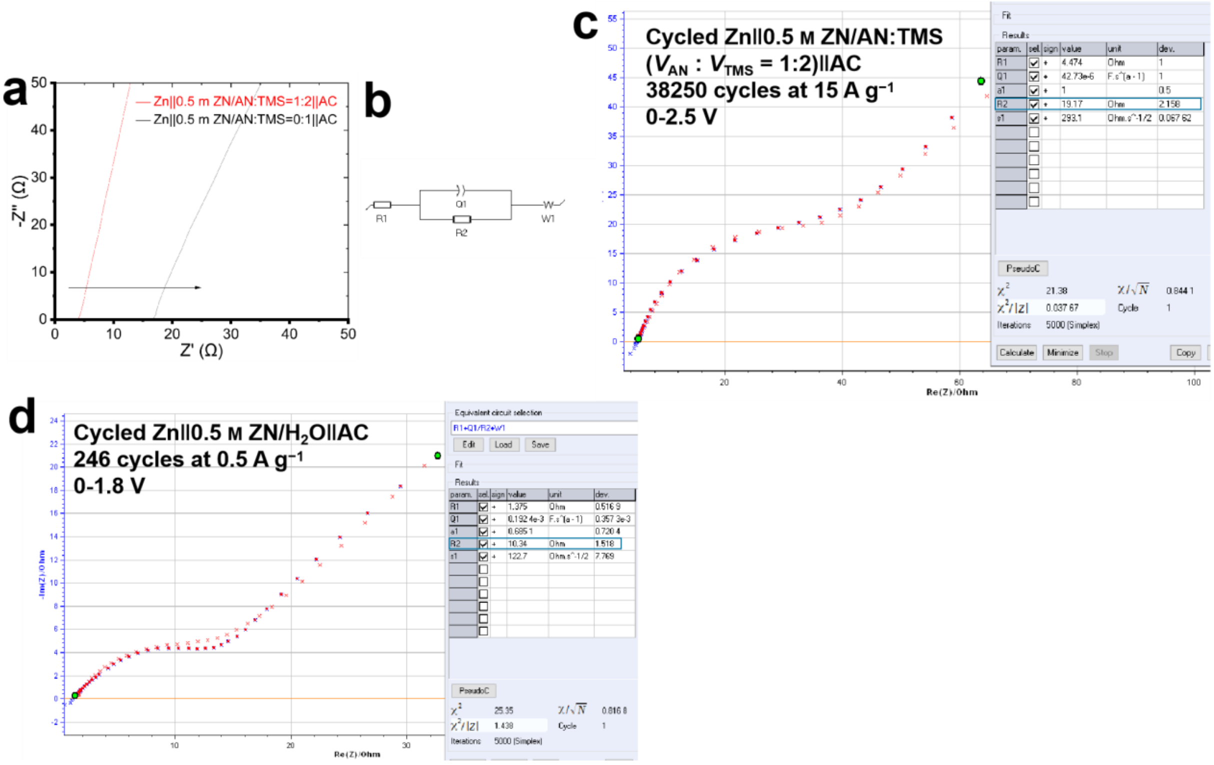

Figure S8. (a) The EIS comparison between fresh zinc ion hybrid capacitor after 12 h rest using 0.5 M Zn[NTf$_2$]$_2$/(V_{AN}:V_{TMS} = 1:2) and 0.5 M Zn[NTf$_2$]$_2$/TMS electrolyte. **(b)** The equivalent circuit model of Zn||AC ion capacitor for Figure S8c, d. R1: the contact resistance. R2: the charge transfer resistance. Q1: a constant phase element. W1: the Warburg diffusion element. **(c, d)** The EIS comparison between cycled Zn||AC ion capacitor under different conditions and using different electrolyte (The blue points are the raw data and the red points are the fitted data).

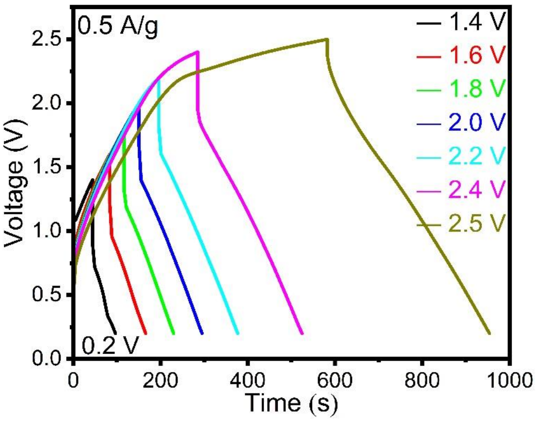

Figure S9. Charge/discharge profiles with different positive cell voltage limits (discharge to 0.2 V).

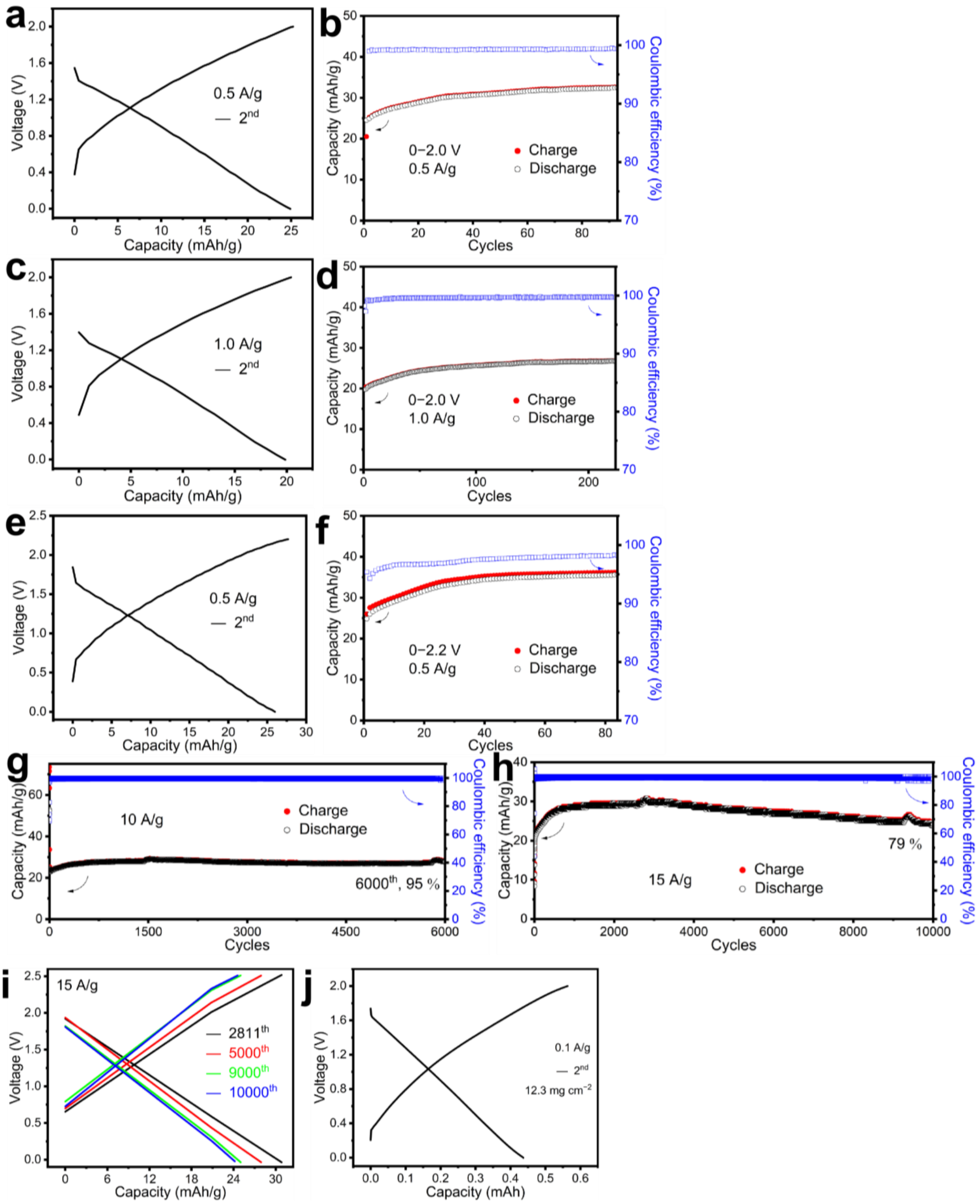

Figure S10. Long-term cycling and corresponding selected charge/discharge profiles. **(a, b)** At 0.5 A g^{-1} between 0 and 2.0 V. **(c, d)** At 1.0 A g^{-1} between 0 and 2.0 V. **(e, f)** At 0.5 A g^{-1} between 0 and 2.2 V. **(g)** long-term cycling at 10 A g^{-1} (activation under 0.5 A g^{-1} for the first three cycles) and **(h, i)** at 15 A g^{-1}. The CE in the curves could be influenced by external temperature influences. When the cell was operated at a higher current rate, the CE values can approach 100%. When the cell was operated at a higher current rate, the CE values can approach 100%. **(j)** Cycling performance at 0.1 A g^{-1} between 0 and 2.0 V using high loading activated carbon electrode in 0.5 M $Zn[NTf_2]_2/(V_{AN}:V_{TMS} = 1:2)$ electrolyte.

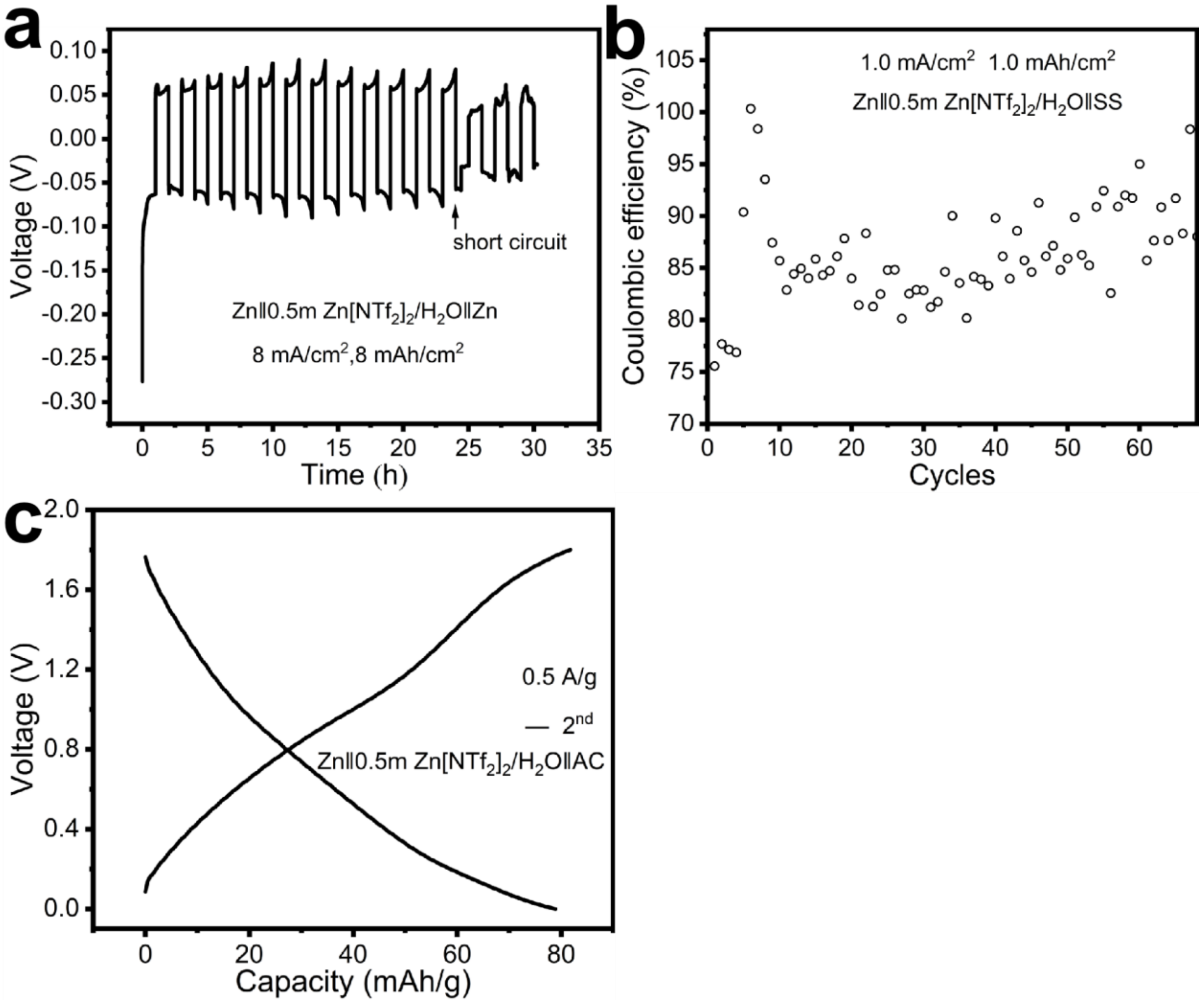

Figure S11. (a) Cycling performance of a symmetric Zn||Zn cell at 8.0 mA cm^{-2} and 8.0 mAh cm^{-2}, **(b)** coulombic efficiency of Zn||SS at 1.0 mA cm^{-2} and 1.0 mAh cm^{-2}, and **(c)** long-term cycling of Zn||AC ion capacitor at 0.5 A g^{-1} between 0 and 1.8 V in a 0.5 M $Zn[NTf_2]_2/H_2O$ electrolyte.

Complete reference of Gaussian 16

Gaussian 16, Revision A.03, M. J. Frisch, G. W. Trucks, H. B. Schlegel, G. E. Scuseria, M. A. Robb, J. R. Cheeseman, G. Scalmani, V. Barone, G. A. Petersson, H. Nakatsuji, X. Li, M. Caricato, A. V. Marenich, J. Bloino, B. G. Janesko, R. Gomperts, B. Mennucci, H. P. Hratchian, J. V. Ortiz, A. F. Izmaylov, J. L. Sonnenberg, D. Williams-Young, F. Ding, F. Lipparini, F. Egidi, J. Goings, B. Peng, A. Petrone, T. Henderson, D. Ranasinghe, V. G. Zakrzewski, J. Gao, N. Rega, G. Zheng, W. Liang, M. Hada, M. Ehara, K. Toyota, R. Fukuda, J. Hasegawa, M. Ishida, T. Nakajima, Y. Honda, O. Kitao, H. Nakai, T. Vreven, K. Throssell, J. A. Montgomery, Jr., J. E. Peralta, F. Ogliaro, M. J. Bearpark, J. J. Heyd, E. N. Brothers, K. N. Kudin, V. N. Staroverov, T. A. Keith, R. Kobayashi, J. Normand, K. Raghavachari, A. P. Rendell, J. C. Burant, S. S. Iyengar, J. Tomasi, M. Cossi, J. M. Millam, M. Klene, C. Adamo, R. Cammi, J.

W. Ochterski, R. L. Martin, K. Morokuma, O. Farkas, J. B. Foresman, and D. J. Fox, Gaussian, Inc., Wallingford CT, 2016.

Table S1. Cartesian coordinates of all the stationary points for Zn−TMS.

Energy = −6976.2766928 Hartree

Center number	Atomic number	Atomic type	Coordinates (angstroms)			Center number	Atomic number	Atomic type	Coordinates (angstroms)		
			X	Y	Z				X	Y	Z
1	30	0	−0.08135	0.096788	−0.18005	35	1	0	−0.79105	−0.94268	−5.14463
2	6	0	1.931091	−0.84845	4.450297	36	1	0	−0.31737	0.769342	−5.39659
3	6	0	1.67203	−2.3561	4.333778	37	1	0	0.943174	−0.4833	−5.14954
4	6	0	0.6886	−2.58952	3.186821	38	7	0	2.070073	0.017218	−0.38349
5	6	0	0.567342	−0.18153	4.382849	39	16	0	2.864831	−1.40157	−0.2024
6	1	0	2.447148	−0.59496	5.386796	40	16	0	2.914173	1.378237	−0.67869
7	1	0	2.560098	−0.49733	3.616691	41	8	0	1.855824	−2.41297	0.07053
8	1	0	1.239859	−2.72539	5.276424	42	8	0	4.052001	−1.3042	0.631046
9	1	0	2.60293	−2.91297	4.158667	43	8	0	4.215277	1.10293	−1.27195
10	1	0	1.163521	−2.72694	2.205793	44	8	0	2.011773	2.335253	−1.29849
11	1	0	−0.04679	−3.38516	3.355422	45	6	0	3.492102	−1.83001	−1.93221
12	1	0	0.563368	0.888326	4.137273	46	6	0	3.293925	2.083069	1.009758
13	1	0	−0.04417	−0.37165	5.277528	47	9	0	4.788667	−1.62472	−2.01198
14	16	0	−0.24007	−1.04273	3.026179	48	9	0	2.871418	−1.11195	−2.85267
15	8	0	0.249934	−0.34731	1.80044	49	9	0	2.230294	2.03705	1.78663
16	8	0	−1.68203	−1.17797	3.215941	50	9	0	4.27246	1.400111	1.574248
17	6	0	−1.70225	4.844551	−1.83217	51	9	0	3.675897	3.337494	0.850144
18	6	0	−2.78252	4.55142	−0.78382	52	9	0	3.241036	−3.10939	−2.14217
19	6	0	−2.0787	4.160167	0.513467	53	7	0	−1.99549	−0.87535	−0.23312
20	6	0	−0.71109	3.692382	−1.76145	54	16	0	−2.1285	−2.49161	0.00754
21	1	0	−2.12694	4.92579	−2.84259	55	16	0	−3.30231	0.01812	−0.64871
22	1	0	−1.19219	5.795947	−1.61054	56	8	0	−3.4783	−2.92259	0.322748
23	1	0	−3.40564	3.711539	−1.12302	57	8	0	−1.00702	−2.91706	0.825171
24	1	0	−3.43749	5.418218	−0.61989	58	8	0	−2.81013	1.267765	−1.21163
25	1	0	−1.80188	5.022065	1.136304	59	8	0	−4.34347	−0.72655	−1.33287
26	1	0	−2.60167	3.413759	1.121947	60	6	0	−1.76493	−3.18564	−1.68327
27	1	0	0.2834	3.870085	−2.18692	61	6	0	−4.04038	0.506901	0.989689
28	1	0	−1.13596	2.753314	−2.14148	62	9	0	−0.50977	−2.93694	−2.01271
29	16	0	−0.48876	3.456981	0.00788	63	9	0	−2.56367	−2.6496	−2.58794
30	8	0	0.5949	4.290335	0.523407	64	9	0	−4.48088	−0.54688	1.641752
31	8	0	−0.45662	2.009608	0.372989	65	9	0	−3.13938	1.137347	1.724095
32	7	0	−0.16578	0.221005	−2.30148	66	9	0	−5.04925	1.328135	0.744758
33	6	0	−0.12314	0.056126	−3.44193	67	9	0	−1.95764	−4.49184	−1.64541
34	6	0	−0.06927	−0.16139	−4.86779						

Table S2. Cartesian coordinates of all the stationary points for Zn.

Energy = −7681.8680975 Hartree

Center number	Atomic number	Atomic type	Coordinates (angstroms)			Center number	Atomic number	Atomic type	Coordinates (angstroms)		
			X	Y	Z				X	Y	Z
1	30	0	−0.10616	0.219812	0.167892	42	1	0	2.318566	−3.70728	−1.50397
2	6	0	3.24319	0.711944	3.98614	43	1	0	2.061037	−3.01136	0.129515
3	6	0	2.346601	0.444835	5.197468	44	16	0	0.175412	−2.74267	−1.23589
4	6	0	1.043035	−0.1192	4.652462	45	8	0	0.304083	−2.19335	−2.58341
5	6	0	2.41411	1.481035	2.966799	46	8	0	−0.19788	−1.78674	−0.13541
6	1	0	4.138339	1.289842	4.256575	47	7	0	−0.91344	0.640829	−1.64527

7	1	0	3.575575	−0.23661	3.537719	48	6	0	−1.47076	0.520267	−2.64762
8	1	0	2.152612	1.381865	5.744148	49	6	0	−2.17531	0.341455	−3.88771
9	1	0	2.803639	−0.26541	5.900899	50	1	0	−3.0338	−0.32277	−3.70633
10	1	0	1.140876	−1.16115	4.315647	51	1	0	−2.52771	1.312638	−4.26313
11	1	0	0.162405	−0.00981	5.300197	52	1	0	−1.50139	−0.12069	−4.624
12	1	0	2.719709	1.343096	1.924163	53	7	0	3.674567	−0.6907	−1.15328
13	1	0	2.328736	2.554786	3.186832	54	16	0	4.227962	−0.75505	0.344052
14	16	0	0.732676	0.850997	3.168363	55	16	0	4.300166	0.250769	−2.29761
15	8	0	0.390943	−0.1378	2.084526	56	8	0	3.189057	−1.34473	1.188393
16	8	0	−0.22649	1.930209	3.409495	57	8	0	4.926008	0.430157	0.833862
17	6	0	−0.80679	5.253783	−1.06154	58	8	0	5.660559	0.718675	−2.063
18	6	0	−1.91544	4.576398	−0.24991	59	8	0	3.947584	−0.30802	−3.59398
19	6	0	−1.28957	4.042592	1.033408	60	6	0	5.52954	−2.07663	0.265438
20	6	0	0.355019	4.272436	−1.09658	61	6	0	3.246589	1.775825	−2.16844
21	1	0	−1.13364	5.496488	−2.08253	62	9	0	6.479002	−1.75118	−0.59508
22	1	0	−0.49383	6.192682	−0.57674	63	9	0	4.9902	−3.22863	−0.10646
23	1	0	−2.34205	3.739088	−0.82288	64	9	0	1.972659	1.471124	−2.37834
24	1	0	−2.73467	5.269661	−0.01275	65	9	0	3.360053	2.332945	−0.97225
25	1	0	−1.1795	4.809304	1.814088	66	9	0	3.63145	2.651373	−3.08572
26	1	0	−1.77106	3.146616	1.445901	67	9	0	6.066949	−2.22413	1.467478
27	1	0	1.345873	4.697283	−1.30839	68	7	0	−3.9063	−0.5705	−0.23295
28	1	0	0.161958	3.417416	−1.75999	69	16	0	−4.75131	−1.91377	−0.44572
29	16	0	0.403842	3.617776	0.579378	70	16	0	−4.49451	0.772949	0.408724
30	8	0	1.381124	4.326015	1.403077	71	8	0	−4.05028	−2.7373	−1.42723
31	8	0	0.577162	2.131818	0.524212	72	8	0	−6.1957	−1.75764	−0.56138
32	6	0	1.087062	−4.99776	−0.1741	73	8	0	−5.67071	0.62376	1.256242
33	6	0	−0.42666	−4.88606	0.067949	74	8	0	−3.3739	1.574724	0.888495
34	6	0	−0.98919	−4.09003	−1.09703	75	6	0	−4.50182	−2.8182	1.158294
35	6	0	1.624061	−3.64523	−0.65567	76	6	0	−5.11066	1.707328	−1.07429
36	1	0	1.616157	−5.31858	0.733687	77	9	0	−4.96577	−2.10912	2.173324
37	1	0	1.270005	−5.75305	−0.9529	78	9	0	−3.21221	−3.05625	1.359124
38	1	0	−0.63936	−4.36298	1.013514	79	9	0	−4.11217	1.968149	−1.91203
39	1	0	−0.8975	−5.87773	0.12211	80	9	0	−6.03095	1.010639	−1.71881
40	1	0	−0.92437	−4.63622	−2.05165	81	9	0	−5.63705	2.855821	−0.67573
41	1	0	−1.99379	−3.65731	−0.97897	82	9	0	−5.14663	−3.97462	1.108967

Table S3. Cartesian coordinates of all the stationary points for Zn−AN.

Energy = −7549.1985098 Hartree

Center number	Atomic number	Atomic type	Coordinates (angstroms)			Center number	Atomic number	Atomic type	Coordinates (angstroms)		
			X	Y	Z				X	Y	Z
1	30	0	−0.69103	0.488434	0.512771	39	1	0	1.902997	−5.17469	2.244237
2	6	0	1.838846	3.998665	2.137218	40	1	0	2.772645	−3.12754	1.220597
3	6	0	1.332175	4.457022	3.507986	41	1	0	1.170639	−3.25826	0.40725
4	6	0	0.124732	3.595462	3.866744	42	1	0	1.219181	−1.57377	4.509585
5	6	0	0.620506	3.913609	1.233272	43	1	0	−0.2518	−2.36589	3.835001
6	1	0	2.577591	4.69744	1.719502	44	16	0	1.21798	−1.63832	2.137985
7	1	0	2.317687	3.008346	2.208268	45	8	0	2.322339	−0.68146	2.180773
8	1	0	1.029588	5.514412	3.453178	46	8	0	−0.07513	−1.17589	1.531965
9	1	0	2.105974	4.369273	4.283403	47	7	0	3.539466	1.013709	−0.81078
10	1	0	0.397905	2.640755	4.337636	48	16	0	4.925099	0.425562	−1.38716
11	1	0	−0.6482	4.099408	4.462428	49	16	0	2.165044	0.258524	−1.03285
12	1	0	0.733008	3.295482	0.333937	50	8	0	5.877451	1.518209	−1.48997
13	1	0	0.213422	4.901203	0.967926	51	8	0	4.7599	−0.51028	−2.49546
14	16	0	−0.62873	3.154331	2.283361	52	8	0	2.157188	−1.19231	−0.93285
15	8	0	−0.51185	1.660284	2.181083	53	8	0	1.170678	1.013215	−0.23083
16	8	0	−1.96102	3.733077	2.106095	54	6	0	5.572773	−0.59972	0.021857
17	6	0	−4.58023	2.240106	−2.53224	55	6	0	1.658864	0.638602	−2.81747
18	6	0	−5.02446	2.089267	−1.07244	56	9	0	4.837244	−1.68291	0.198874
19	6	0	−4.04499	2.859815	−0.1886	57	9	0	5.583408	0.107234	1.139019
20	6	0	−3.08235	1.986934	−2.54454	58	9	0	2.587094	1.368641	−3.40953

21	1	0	−5.09769	1.528154	−3.19071	59	9	0	1.509093	−0.49308	−3.47586
22	1	0	−4.79289	3.256258	−2.90231	60	9	0	0.520416	1.305747	−2.82511
23	1	0	−5.00954	1.025746	−0.79178	61	9	0	6.814091	−0.96714	−0.2662
24	1	0	−6.04766	2.459547	−0.91821	62	7	0	−3.0089	−2.17015	−0.68968
25	1	0	−4.31763	3.914814	−0.04466	63	16	0	−3.50378	−1.36327	0.580998
26	1	0	−3.82836	2.401818	0.784156	64	16	0	−1.54998	−2.10237	−1.3283
27	1	0	−2.53223	2.343305	−3.42643	65	8	0	−4.94074	−1.17842	0.545398
28	1	0	−2.83316	0.936689	−2.33948	66	8	0	−2.68598	−0.16874	0.902091
29	16	0	−2.50098	2.922966	−1.12888	67	8	0	−0.9335	−0.75435	−1.23417
30	8	0	−2.17515	4.298065	−1.50076	68	8	0	−0.71619	−3.25564	−1.04233
31	8	0	−1.40596	2.169376	−0.45154	69	6	0	−3.20289	−2.54029	2.005486
32	6	0	1.517184	−3.66313	3.785781	70	6	0	−1.9942	−2.25201	−3.12535
33	6	0	1.301173	−4.2672	2.394917	71	9	0	−2.11679	−3.25446	1.783155
34	6	0	1.687198	−3.20444	1.374289	72	9	0	−4.23973	−3.34606	2.119611
35	6	0	0.84374	−2.29891	3.774684	73	9	0	−2.57361	−3.41605	−3.3355
36	1	0	1.092446	−4.29383	4.579567	74	9	0	−2.8114	−1.27972	−3.48111
37	1	0	2.594656	−3.54942	3.987569	75	9	0	−0.88093	−2.17413	−3.8277
38	1	0	0.242654	−4.54238	2.265977	76	9	0	−3.05708	−1.84668	3.118705

Table S4. Cartesian coordinates of all the stationary points for Zn−[NTf_2]$^-$.

Energy = −5855.1498239 Hartree

Center number	Atomic number	Atomic type	Coordinates (angstroms)			Center number	Atomic number	Atomic type	Coordinates (angstroms)		
			X	Y	Z				X	Y	Z
1	30	0	0.01796	−0.36652	−0.68725	35	6	0	4.522792	−1.0595	−1.92937
2	6	0	−0.13097	−1.57715	4.214972	36	1	0	6.563331	−1.13905	−1.20879
3	6	0	0.129329	−3.08611	4.189004	37	1	0	5.709305	−2.69777	−1.16734
4	6	0	0.680104	−3.39634	2.805928	38	1	0	5.184229	−0.25709	0.633015
5	6	0	−0.88754	−1.20628	2.942428	39	1	0	5.71861	−1.86346	1.172295
6	1	0	−0.70926	−1.27619	5.099741	40	1	0	3.534552	−2.84507	0.740972
7	1	0	0.828572	−1.03794	4.240753	41	1	0	3.027209	−1.17322	1.191499
8	1	0	−0.80612	−3.64409	4.356582	42	1	0	4.516186	−1.45628	−2.95388
9	1	0	0.845833	−3.39242	4.964025	43	1	0	4.507696	0.040092	−1.94601
10	1	0	1.700424	−3.01072	2.663642	44	16	0	2.987492	−1.57588	−1.14907
11	1	0	0.619488	−4.44485	2.483301	45	8	0	2.51066	−2.84733	−1.69173
12	1	0	−0.65184	−0.21441	2.539451	46	8	0	2.038923	−0.41527	−1.20387
13	1	0	−1.97827	−1.32192	3.017331	47	7	0	−0.53984	−1.10595	−2.49224
14	16	0	−0.38674	−2.44439	1.721249	48	6	0	−0.69954	−1.54928	−3.54478
15	8	0	0.471816	−1.8334	0.647504	49	6	0	−0.89639	−2.10901	−4.85499
16	8	0	−1.52456	−3.24733	1.270173	50	1	0	0.009716	−2.65754	−5.15261
17	6	0	−5.24259	−0.76175	−1.72092	51	1	0	−1.75415	−2.79729	−4.83419
18	6	0	−4.33091	−1.91384	−2.15325	52	1	0	−1.0899	−1.29868	−5.5731
19	6	0	−3.60009	−2.40642	−0.91079	53	7	0	0.137799	1.583079	−0.01218
20	6	0	−4.38373	0.175074	−0.88549	54	16	0	1.331849	2.034611	1.012104
21	1	0	−5.66639	−0.22805	−2.58332	55	16	0	−0.85975	2.730114	−0.63031
22	1	0	−6.0818	−1.14411	−1.11746	56	8	0	1.855713	0.823922	1.623441
23	1	0	−3.60348	−1.55069	−2.89608	57	8	0	0.934706	3.16572	1.833868
24	1	0	−4.89724	−2.73653	−2.61233	58	8	0	−0.16949	3.983461	−0.88507
25	1	0	−4.21101	−3.08175	−0.29457	59	8	0	−1.64237	2.084337	−1.67256
26	1	0	−2.61326	−2.85552	−1.07642	60	6	0	2.707077	2.683753	−0.11353
27	1	0	−4.9261	0.83201	−0.19207	61	6	0	−2.08116	3.106566	0.755473
28	1	0	−3.67758	0.76426	−1.48894	62	9	0	2.403488	2.522248	−1.38414
29	16	0	−3.37852	−0.9343	0.116659	63	9	0	3.818393	2.022563	0.158827
30	8	0	−3.95733	−1.1142	1.448398	64	9	0	−2.10805	2.135677	1.646625
31	8	0	−1.96201	−0.46153	0.083575	65	9	0	−1.75278	4.237024	1.341929
32	6	0	5.59357	−1.61399	−1.00331	66	9	0	−3.27929	3.231664	0.209777
33	6	0	5.111166	−1.33519	0.423978	67	9	0	2.891116	3.964152	0.134588
34	6	0	3.6527	−1.77476	0.520398						

Table S5. Cartesian coordinates of all the stationary points for TMS.

Energy = −705.5872595 Hartree

Center number	Atomic number	Atomic type	Coordinates (angstroms)			Center number	Atomic number	Atomic type	Coordinates (angstroms)		
			X	Y	Z				X	Y	Z
1	6	0	1.784608	0.742673	−0.14368	9	1	0	0.453849	−1.3413	−1.51365
2	6	0	1.754249	−0.76095	0.149121	10	1	0	0.079558	−2.22398	0.011838
3	6	0	0.441937	−1.27911	−0.41556	11	1	0	0.114674	2.209651	−0.23114
4	6	0	0.449002	1.315292	0.311622	12	1	0	0.412411	1.498715	1.395007
5	1	0	2.619044	1.244633	0.367122	13	16	0	−0.76919	0.001267	0.003396
6	1	0	1.911254	0.904935	−1.22636	14	8	0	−1.5973	0.320338	−1.16641
7	1	0	1.790309	−0.93565	1.237077	15	8	0	−1.43559	−0.34539	1.266132
8	1	0	2.610281	−1.2843	−0.30103						

Table S6. Cartesian coordinates of all the stationary points for AN.

Energy = −132.662273 Hartree

Center number	Atomic number	Atomic type	Coordinates (angstroms)			Center number	Atomic number	Atomic type	Coordinates (angstroms)		
			X	Y	Z				X	Y	Z
1	6	0	0.27504	0.000173	−6E−06	4	1	0	−1.54827	1.015876	−0.19169
2	7	0	1.433108	−5.3E−05	0	5	1	0	−1.54743	−0.67439	−0.78403
3	6	0	−1.17314	0.000019	−1.3E−05	6	1	0	−1.54749	−0.34227	0.975839

Table S7. Cartesian coordinates of all the stationary points for $[NTf_2]^-$.

Energy = −1826.7175241 Hartree

Center number	Atomic number	Atomic type	Coordinates (angstroms)			Center number	Atomic number	Atomic type	Coordinates (angstroms)		
			X	Y	Z				X	Y	Z
1	7	0	−1.2E−05	−0.00018	0.858751	9	6	0	−2.51185	0.383749	−0.05375
2	16	0	1.1323	0.847673	0.106674	10	9	0	2.122822	−1.44278	−0.74728
3	16	0	−1.13243	−0.84787	0.106671	11	9	0	2.916814	−0.78419	1.142992
4	8	0	1.656971	1.838837	1.036525	12	9	0	−2.91961	0.780919	1.143143
5	8	0	0.836088	1.247144	−1.26494	13	9	0	−2.12113	1.44493	−0.74333
6	8	0	−0.83624	−1.24739	−1.26493	14	9	0	−3.53312	−0.18233	−0.68454
7	8	0	−1.65725	−1.83897	1.036492	15	9	0	3.534781	0.18419	−0.68044
8	6	0	2.511936	−0.38362	−0.05374						
